# Endocrine and Molecular Mechanisms of Oocyte Aging: From Gonadotropin Signaling to Metabolic and Microenvironmental Dysfunction

**DOI:** 10.3390/cimb48090959

**Published:** 2026-09-19

**Authors:** Maria Fotara, Efthalia Moustakli, Maria Anastasia Daskalaki, Despoina Mavrogianni, Ioannis Arkoulis, Spyridon Topis, Anastasios Potiris, Konstantinos Louis, Nikolaos Kathopoulis, Ekaterini Domali, George Daskalakis, Sofoklis Stavros

**Affiliations:** 1 First Department of Obstetrics and Gynecology, Alexandra Hospital, Medical School, National and Kapodistrian University of Athens, 11528 Athens, Greece; mr.fotara@gmail.com (M.F.); anastasia.daskalaki00@gmail.com (M.A.D.); dmavrogianni@med.uoa.gr (D.M.); kdomali@yahoo.fr (E.D.); gdaskalakis@yahoo.com (G.D.); 2 Department of Nursing, School of Health Sciences, University of Ioannina, 45500 Ioannina, Greece; ef.moustakli@uoi.gr; 3 Third Department of Obstetrics and Gynecology, University General Hospital “ATTIKON”, Medical School, National and Kapodistrian University of Athens, 12462 Athens, Greece; garkoylis@hotmail.com (I.A.); spyros.topis1996@gmail.com (S.T.); apotiris@med.uoa.gr (A.P.); kostaslouisss@gmail.com (K.L.); nickatho@gmail.com (N.K.)

**Keywords:** oocyte aging, mitochondrial dysfunction, telomeres, epigenetic modifications, ovarian follicular microenvironment, cumulus cells, female fertility

## Abstract

Ovarian aging and oocyte aging are closely interconnected but distinct components of reproductive aging. Whereas ovarian aging encompasses progressive follicular depletion and changes in ovarian endocrine and somatic-cell function, oocyte aging refers more specifically to the age-associated deterioration in the molecular, cellular, and developmental competence of the oocyte. This deterioration contributes to increased aneuploidy, impaired embryonic development, implantation failure, and early pregnancy loss. This narrative review synthesizes current evidence on the endocrine, cellular, and molecular mechanisms underlying oocyte aging and their potential interactions, and examines emerging strategies for preserving oocyte competence. Age-related dysregulation of the hypothalamic–pituitary–ovarian axis, altered gonadotropin signaling, and changes in intraovarian endocrine and paracrine communication may disrupt follicular development and the follicular microenvironment. These changes may compromise functional interactions between the oocyte and surrounding somatic cells. These alterations may converge with mitochondrial dysfunction, oxidative stress, mtDNA abnormalities, impaired energy metabolism, alterations in telomere biology, and epigenetic dysregulation, ultimately compromising oocyte meiotic competence and developmental potential. Experimental strategies, including antioxidant supplementation, restoration of NAD^+^ metabolism, mitochondrial transfer, and exosome-based interventions, have shown potential to ameliorate features of oocyte aging, primarily in animal and preclinical models. However, none of these interventions has been shown to delay physiological oocyte aging in women, and evidence supporting their clinical effectiveness and long-term safety remains insufficient. Understanding how endocrine dysregulation interacts with metabolic, genomic, and epigenetic alterations and changes in the follicular microenvironment may facilitate the identification of biomarkers and the development of targeted approaches to preserve oocyte quality during reproductive aging.

## 1. Introduction

Ovarian aging constitutes a physiological process characterized by a gradual decline in both the quantity and quality of ovarian follicles and, consequently, oocytes, together with progressive changes in ovarian endocrine function. These qualitative alterations of oocytes are described by the term oocyte aging, a complex and multifactorial process that critically affects female fertility [1]. Although closely interconnected, ovarian aging and oocyte aging represent distinct concepts. Ovarian aging encompasses follicular depletion and changes in ovarian endocrine and somatic-cell function, whereas oocyte aging refers more specifically to the progressive deterioration in the molecular, cellular, and developmental competence of the oocyte with advancing reproductive age [1,2]. Oocyte quality is closely associated with developmental competence and influences the ability of an oocyte to complete meiosis, undergo fertilization, and support successful implantation and normal embryonic development [3]. At birth, the ovaries contain approximately 1–2 million oocytes within primordial follicles, and this finite pool progressively declines throughout life. Oocytes are metabolically active cells that can remain arrested at prophase I of meiosis for decades, during which they are continuously exposed to endogenous and environmental factors. This prolonged lifespan, combined with their limited capacity to dilute accumulated cellular damage through cell division, renders them particularly vulnerable to the accumulation of molecular damage and the gradual loss of their functional capacity [4,5].

Ovarian and oocyte aging occur within a tightly regulated endocrine system. The hypothalamic–pituitary–ovarian axis (HPO) coordinates follicular recruitment, growth, maturation, ovulation, and luteinization through the actions of gonadotropin-releasing hormone, follicle-stimulating hormone (FSH), and luteinizing hormone (LH). Within the ovary, gonadotropin signaling regulates granulosa- and theca-cell function, steroidogenesis, and the production of intraovarian mediators that support oocyte development [1]. As the follicular pool diminishes, circulating concentrations of anti-Müllerian hormone (AMH) and inhibin B decline, while the progressive loss of ovarian negative feedback contributes to increased FSH concentrations. These endocrine changes reflect declining ovarian reserve but may also alter the follicular environment in which the oocyte develops. Moreover, age-related changes in gonadotropin responsiveness and endocrine–metabolic signaling may affect mitochondrial activity, redox homeostasis, and communication between the oocyte and its surrounding somatic cells [6].

Although the complete sequence of events responsible for the age-related decline in oocyte quality has not been fully elucidated, several interconnected endocrine, cellular, and molecular mechanisms appear to play crucial roles in this process [7]. Specifically, oocyte aging is characterized by progressive mitochondrial dysfunction, increased production of reactive oxygen species (ROS) and oxidative stress, alterations in telomere biology, epigenetic alterations, and changes in the follicular microenvironment and bidirectional communication between the oocyte and surrounding granulosa and cumulus cells [1,8,9]. Rather than occurring independently, these alterations may reinforce one another: endocrine dysregulation can affect follicular-cell metabolism and oocyte support, while mitochondrial and oxidative damage may further impair hormone-responsive signaling pathways. Furthermore, environmental factors and lifestyle, such as obesity, metabolic dysfunction, dietary patterns, and exposure to endocrine-disrupting chemicals, may exacerbate these mechanisms and accelerate reproductive aging [10,11].

Understanding the endocrine and molecular mechanisms underlying oocyte aging is essential for advancing reproductive biology and assisted reproductive medicine. Recent studies have investigated several strategies aimed at preserving or restoring oocyte competence, including antioxidant supplementation, restoration of NAD^+^ metabolism, mitochondrial transfer, and exosome-based modulation of the follicular microenvironment [12,13,14,15]. Although these approaches have produced encouraging findings, most supporting evidence remains preclinical, and their safety and effectiveness in humans have not been established. Although previous reviews have examined individual aspects of reproductive aging, including mitochondrial dysfunction, oxidative stress, epigenetic alterations, and ovarian endocrine changes, these processes are increasingly recognized as interconnected components of the aging follicular environment rather than isolated mechanisms. An integrated evaluation is therefore needed to clarify how systemic endocrine changes, ovarian somatic-cell dysfunction, and oocyte-intrinsic molecular alterations collectively contribute to declining developmental competence. Such integration is particularly important because direct mechanistic evidence from human oocytes remains limited, with many proposed pathways supported predominantly by findings from granulosa or cumulus cells, ovarian tissue, embryos, or experimental animal models [16,17]. Accordingly, throughout this review, evidence derived directly from human oocytes is distinguished from findings obtained from ovarian somatic cells and experimental models, and associative observations are differentiated from mechanistic evidence.

## 2. Literature Search Strategy and Study Selection

This narrative review aimed to integrate and critically analyze current evidence on the endocrine, cellular, and molecular mechanisms involved in oocyte aging. The structured literature search was conducted using PubMed and Google Scholar and covered publications from 1 January 2017 to 31 December 2025. PubMed was used as the principal bibliographic database because of its broad coverage of biomedical and reproductive research, while Google Scholar was used as a complementary source to identify additional relevant literature. Given the narrative rather than systematic design of the review, additional bibliographic databases were not systematically searched. The literature was subsequently updated through targeted searches during manuscript preparation, with the databases last queried on 17 August 2026. These targeted searches were used to identify relevant studies published in 2026, while earlier studies were also considered when necessary to provide foundational evidence or essential mechanistic context.

The search strategy employed combinations of relevant keywords in English, including “oocyte aging,” “ovarian aging,” “gonadotropin signaling,” “follicle-stimulating hormone,” “luteinizing hormone,” “anti-Müllerian hormone,” “steroidogenesis,” “mitochondrial DNA in oocytes,” “oxidative stress,” “reactive oxygen species,” “telomeres in oocytes,” “epigenetic mechanisms,” “oocyte microenvironment,” “granulosa cells,” “cumulus cells,” “NAD^+^,” “mitochondrial transfer,” and “exosomes.” Search terms were combined according to the thematic domain under investigation rather than applied as a single standardized search string. Representative PubMed search strategies reflecting the domain-specific combinations used during the literature search are provided in Table A1. Relevant reference lists were also examined to identify additional studies not retrieved through the initial searches.

Eligible publications included English-language original studies examining human oocytes, ovarian follicles, granulosa or cumulus cells, and experimental models of reproductive aging. Rodent studies were included when they provided mechanistic or experimental evidence that could not feasibly be obtained from human oocytes or other human reproductive material. Mechanistically relevant review articles were used to contextualize the findings and identify additional primary studies. Conference abstracts, opinion articles, non-peer-reviewed publications, and descriptive reports lacking sufficient mechanistic evidence were excluded. Article selection was based on relevance to the predefined thematic scope and the extent to which individual studies provided interpretable mechanistic or translational evidence rather than free full-text availability. Given the narrative design, study selection was not based on a formal quality-scoring threshold.

The retrieved publications were independently assessed by two authors based on title and abstract, followed by full-text evaluation of potentially relevant articles. Disagreements regarding inclusion were resolved through discussion and consensus. Given the narrative design of the review, the literature search was intended to provide a structured but non-exhaustive identification of relevant evidence rather than to define a closed systematic-review evidence set. The available evidence was synthesized narratively according to the principal mechanisms underlying oocyte aging, including gonadotropin and intraovarian signaling, mitochondrial and metabolic dysfunction, oxidative stress, alterations in telomere biology, epigenetic dysregulation, alterations in the follicular microenvironment, and emerging therapeutic strategies.

During synthesis, particular consideration was given to the experimental model and biological material examined, distinguishing evidence derived directly from human oocytes from findings obtained from human ovarian somatic cells, ovarian tissue, embryos, and experimental animal models. Findings were interpreted according to the strength and directness of the available evidence, with particular caution applied when extrapolating mechanistic observations from animal models or surrogate human tissues to physiological oocyte aging in humans. Because this was a narrative review, no meta-analysis or formal risk-of-bias assessment was performed.

## 3. Endocrine and Molecular Mechanisms of Oocyte Aging

### 3.1. Gonadotropin and Intraovarian Signaling

Reproductive aging is characterized by progressive remodeling of the HPO axis and the intraovarian signaling pathways that regulate follicular development [18,19]. Through their actions on ovarian somatic cells, FSH and LH coordinate key processes involved in folliculogenesis, steroidogenesis, oocyte maturation, ovulation, and corpus luteum development. Systemic gonadotropin signaling is integrated with the local follicular microenvironment through locally secreted regulators such as AMH, inhibins, activins, and oocyte-derived growth factors [20].

An increase in circulating FSH levels is one of the earliest endocrine alterations observed during reproductive aging. This increase is closely associated with the progressive depletion of the follicular pool, which results in reduced ovarian inhibin B secretion. As the follicular reserve declines, reduced ovarian negative feedback on the pituitary gland increases FSH secretion, initially helping to maintain follicular recruitment and estrogen production. AMH levels progressively decline as the population of preantral and small antral follicles decreases, whereas more pronounced changes in estradiol and LH generally occur later in the menopausal transition. Therefore, elevated FSH and reduced AMH and inhibin B primarily reflect depletion of the follicular pool rather than direct measures of declining oocyte quality [6,21,22].

Notably, reproductive aging involves not only changes in circulating hormone levels but also potential alterations in the responsiveness of ovarian somatic cells to gonadotropin. FSHR is expressed by granulosa cells, and FSH signaling promotes granulosa-cell proliferation, aromatase activity, estradiol production, and functional differentiation required for follicular maturation. Human studies have reported age-related alterations in this signaling milieu [23,24,25]. In a study by Wu et al., granulosa cells isolated from young oocyte donors and younger and older women undergoing IVF showed reduced expression of FSHR, CYP19A1 (aromatase), and HSD17B, together with increased expression of LHCGR, CYP11A1, and the progesterone receptor. These changes were associated with reduced granulosa-cell proliferation and increased apoptosis and were interpreted as evidence of age-associated premature luteinization. However, because the study involved women undergoing controlled ovarian hyperstimulation (COH), with the older group representing an infertility population, it remains difficult to distinguish physiological age-related changes from effects associated with infertility or exogenous gonadotropin exposure [23].

Age-associated changes in receptor regulation have also been reported in human preovulatory follicles. Regan et al. reported that the relationships among ovarian reserve, FSHR expression, and granulosa-cell apoptosis differed between younger and older women undergoing IVF [24]. In younger women, granulosa-cell apoptosis was negatively correlated with FSHR and BMPR1B expression; however, these relationships were altered with advancing age. A subsequent study by the same group showed that growth hormone co-treatment altered FSHR, LHR, BMPR1B, and GHR expression in granulosa cells from older women with diminished ovarian reserve. However, although these findings indicate age-associated changes in receptor regulation, they do not establish impaired FSH or LH signaling as a primary driver of human oocyte aging. Moreover, receptor expression alone does not establish altered downstream signaling activity or functional responsiveness [24,25]. Accordingly, studies of luteinized granulosa cells collected after controlled ovarian stimulation can identify age-associated differences in receptor expression and somatic-cell phenotype under gonadotropin-exposed ART conditions, but they cannot establish that the same alterations occur in unstimulated follicles or demonstrate impaired gonadotropin signaling within the oocyte itself.

Modifications in gonadotropin signal transduction should thus be considered in relation to the entire intraovarian regulatory network. The age-associated decline in AMH and inhibin B primarily reflects depletion and altered function of the growing follicular population; however, these changes also modify the endocrine environment in which the remaining follicles develop. In older women, the combination of a declining antral follicle count, reduced inhibin B, increased FSH, and decreased AMH production per follicle suggests progressive remodeling of both pituitary and intraovarian regulatory systems as menopause approaches. Oocyte-derived factors, such as growth differentiation factor 9 (GDF9) and bone morphogenetic protein 15 (BMP15), interact with granulosa-cell signaling pathways to regulate follicular growth and differentiation. However, whether age-associated alterations in these local signaling networks directly impair human oocyte competence remains incompletely defined [20].

Consequently, the connection between endocrine reproductive aging and oocyte quality cannot be inferred solely from increases or decreases in individual circulating reproductive hormones. Reproductive aging appears to involve a progressive loss of coordination among systemic gonadotropin signaling, ovarian receptor responsiveness, steroidogenesis, and local oocyte–somatic cell interactions. Importantly, elevated FSH and reduced AMH are well-established markers of declining ovarian reserve, whereas evidence that these hormonal changes directly contribute to molecular alterations in human oocytes remains limited [18,26].

Such endocrine remodeling may also intersect with the cellular and metabolic processes discussed in the following sections. Granulosa and cumulus cells provide metabolic substrates and paracrine signals necessary for oocyte maturation; thus, altered gonadotropin responsiveness may influence somatic-cell steroidogenesis, metabolism, and mitochondrial activity [27,28]. In turn, impaired somatic-cell function may disrupt redox homeostasis, metabolic support, and bidirectional communication with the oocyte, potentially affecting meiotic and developmental competence. This sequence provides a biologically plausible link between age-associated endocrine remodeling and the mitochondrial, redox, and cellular alterations discussed below; however, it has not been established as a causal pathway of human oocyte aging. Taken together, current evidence supports viewing gonadotropin and intraovarian signaling as components of an integrated follicular network rather than as independent determinants of oocyte aging [29,30].

### 3.2. Mitochondrial Dysfunction, Energy Metabolism, and Oxidative Stress

Mitochondria are fundamental organelles that play a central role in oocyte cytoplasmic maturation and the maintenance of developmental competence by regulating ATP production, cellular metabolism, calcium homeostasis, and redox signaling [31,32]. Under physiological conditions, low levels of ROS are generated and efficiently neutralized by endogenous antioxidant systems, such as glutathione (GSH). At controlled concentrations, ROS also participate in physiological signaling; however, their excessive production or insufficient elimination disrupts redox homeostasis and causes oxidative damage to mitochondrial DNA (mtDNA), membrane lipids, and proteins [33,34]. During oocyte growth, mitochondria and mtDNA accumulate to establish the mitochondrial reserve required for meiotic progression, spindle assembly, fertilization, and early embryonic development. By contrast, mtDNA replication is largely completed by the germinal-vesicle stage and remains limited during meiotic maturation and early preimplantation development [2,32].

Studies examining the relationship between maternal age and mtDNA content in human oocytes and related reproductive material have yielded conflicting results. Reports have described reduced, unchanged, or increased mtDNA content in association with maternal age and oocyte maturation status [8,35,36,37]. This variability may reflect differences in oocyte maturation stage, ovarian stimulation regimens, individual patient characteristics, sample sizes, and methods of mtDNA quantification. Therefore, mtDNA copy number cannot currently be considered a reliable standalone biomarker of oocyte aging or mitochondrial competence.

**Figure 1:**
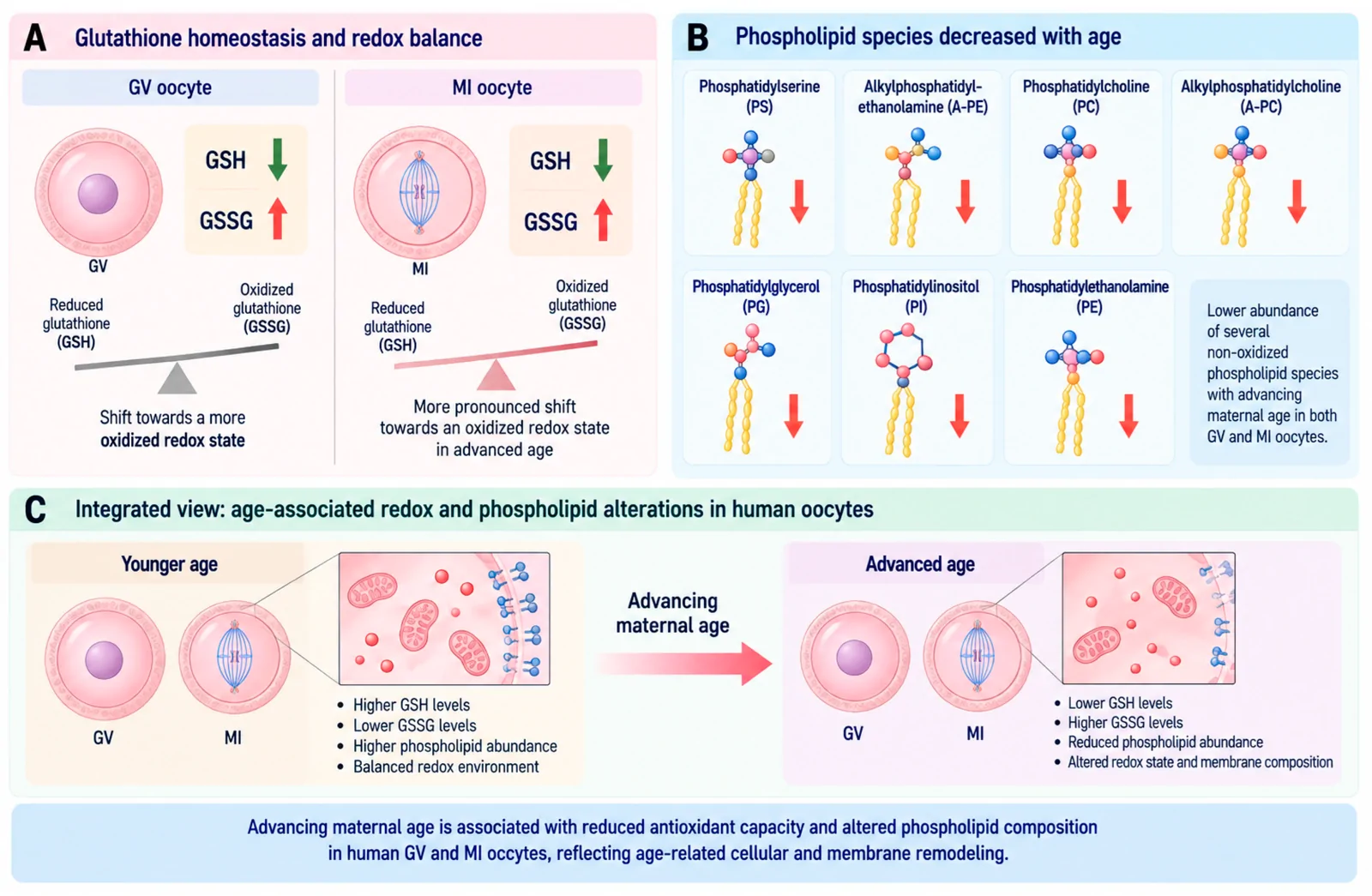
Age-associated alterations in glutathione homeostasis and phospholipid abundance in human GV and MI oocytes. (**A**) Age-associated changes in glutathione homeostasis, characterized by reduced glutathione (GSH) and increased oxidized glutathione (GSSG), with a more pronounced shift toward an oxidized redox state in MI oocytes from women of advanced reproductive age. (**B**) Several non-oxidized phospholipid species, including phosphatidylserine (PS), alkylphosphatidylethanolamine (A-PE), phosphatidylcholine (PC), alkylphosphatidylcholine (A-PC), phosphatidylglycerol (PG), phosphatidylinositol (PI), and phosphatidylethanolamine (PE), showed lower abundance with advancing maternal age. (**C**) Integrated schematic illustrating age-associated alterations in glutathione redox balance and phospholipid composition in human GV and MI oocytes. Figure created by the authors based on the findings of Smits et al. [38].

In addition to possible alterations in mtDNA content, aging is associated with metabolic alterations that may contribute to impaired mitochondrial function and declining oocyte quality. A recent study by Smits et al. (2023) investigated age-associated metabolic and redox alterations in germinal vesicle (GV) and metaphase I (MI) oocytes and cumulus cells obtained from women aged 23–42 years undergoing in vitro fertilization (IVF) [38]. GV and MI oocytes from women aged 35–42 years exhibited reduced glutathione (GSH) levels and increased oxidized glutathione (GSSG), resulting in a lower GSH/GSSG ratio indicative of age-associated redox imbalance (Figure 1). The same study demonstrated reduced levels of several non-oxidized phospholipid species and evidence of lipid peroxidation, supporting an age-associated shift toward oxidative damage in oocytes from women of advanced reproductive age.

**Figure 2:**
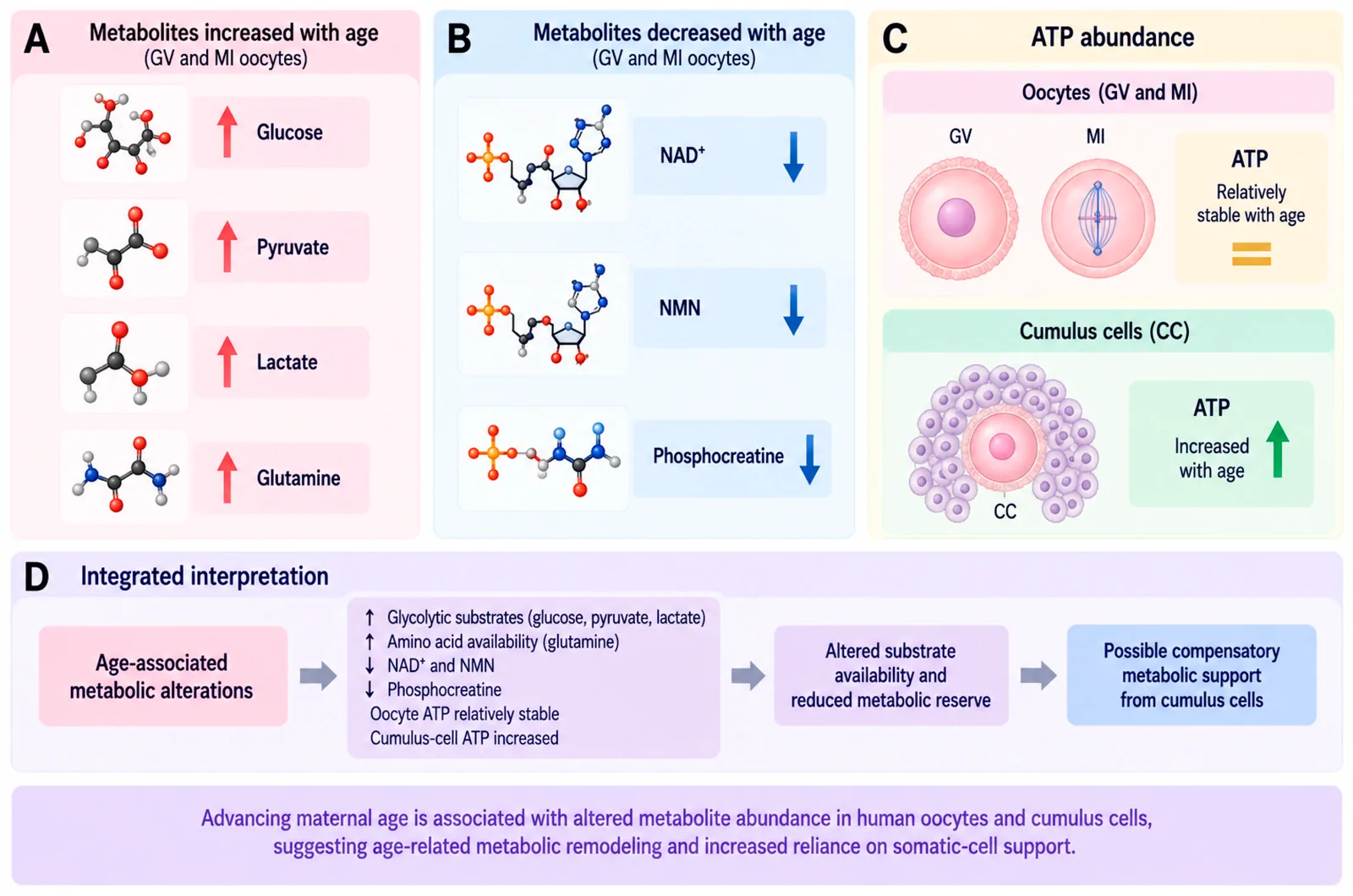
Age-associated metabolic alterations in human GV and MI oocytes and cumulus cells. (**A**) Metabolites showing increased abundance with advancing maternal age in GV and MI oocytes, including glucose, pyruvate, lactate, and glutamine. (**B**) Metabolites showing decreased abundance with advancing maternal age, including NAD^+^, nicotinamide mononucleotide (NMN), and phosphocreatine. (**C**) ATP abundance remained relatively stable across age groups in GV and MI oocytes, whereas ATP abundance increased with age in cumulus cells. (**D**) Integrated schematic illustrating the age-associated metabolic remodeling observed in oocytes and cumulus cells, characterized by altered substrate availability, reduced NAD^+^/NMN and phosphocreatine abundance, and increased cumulus-cell ATP, potentially reflecting compensatory metabolic support from surrounding somatic cells. Figure created by the authors based on the findings of Smits et al. [38].

Smits et al. also reported the accumulation of glycolytic substrates and related metabolites, including glucose, pyruvate, and lactate, in aged oocytes [38]. This pattern suggests altered substrate utilization and a potential compensatory reliance on non-mitochondrial energy pathways rather than a direct increase in glycolytic flux. Reduced levels of NAD^+^, its precursor nicotinamide mononucleotide (NMN), and phosphocreatine were consistent with altered energy metabolism and reduced metabolic reserve. ATP levels were elevated in cumulus cells, whereas they remained relatively stable in GV and MI oocytes. This finding raises the possibility that surrounding somatic cells may increase their metabolic support of aging oocytes (Figure 2). However, metabolite concentrations do not directly demonstrate metabolic flux or substrate transfer, and this interpretation therefore requires further functional investigation. Moreover, because the oocytes were obtained following controlled ovarian stimulation, an influence of exogenous gonadotropin on the observed metabolic profiles cannot be excluded.

Related but distinct findings were reported by Liu et al. (2017), who examined mural granulosa cells rather than cumulus cells [39]. Mitochondria in mural granulosa cells from women of advanced reproductive age exhibited an elongated morphology, numerous cristae, and high-density matrix particles. Alongside these structural alterations, oxidative phosphorylation capacity, ATP levels, ATP5A1 expression, and mitochondrial membrane potential decreased with age, while morphologically abnormal mitochondria became more prevalent. Therefore, mitochondrial elongation should not be interpreted as evidence of increased mitochondrial activity but may instead reflect stress-induced remodeling or an insufficient compensatory response to declining oxidative phosphorylation. These observations suggest that morphological remodeling does not necessarily preserve mitochondrial function in aging follicular somatic cells; however, because these findings derive from mural granulosa cells, they should not be directly extrapolated to the oocyte.

Mitochondrial dysfunction may also involve alterations in mtDNA integrity, including point mutations, deletions, and changes in heteroplasmy. However, whether mtDNA mutations progressively accumulate in human oocytes with maternal age remains unresolved. Yang et al. (2020) reported a greater burden of mtDNA point mutations in oocytes from women aged ≥38 years than in those from women aged ≤30 years [40]. Using POLG-mutator mice, the researchers subsequently demonstrated that experimentally increased mtDNA mutation burden altered the oocyte NADH/NAD^+^ redox state and reduced female fertility, providing mechanistic evidence that mtDNA mutations may contribute to reproductive dysfunction. In contrast, Boucret et al. (2017) detected no significant age-related accumulation of heteroplasmic mtDNA point mutations in 53 immature oocytes obtained from 35 women [41]. A subsequent study employing highly accurate duplex sequencing similarly found no age-associated increase in de novo mtDNA mutations in human oocytes and suggested that purifying selection may limit the transmission of functionally deleterious variants [42]. Differences among studies may arise from variation in cohort composition, oocyte developmental stage, sequencing depth and accuracy, heteroplasmy-detection thresholds, and the genomic regions analyzed.

**Figure 3:**
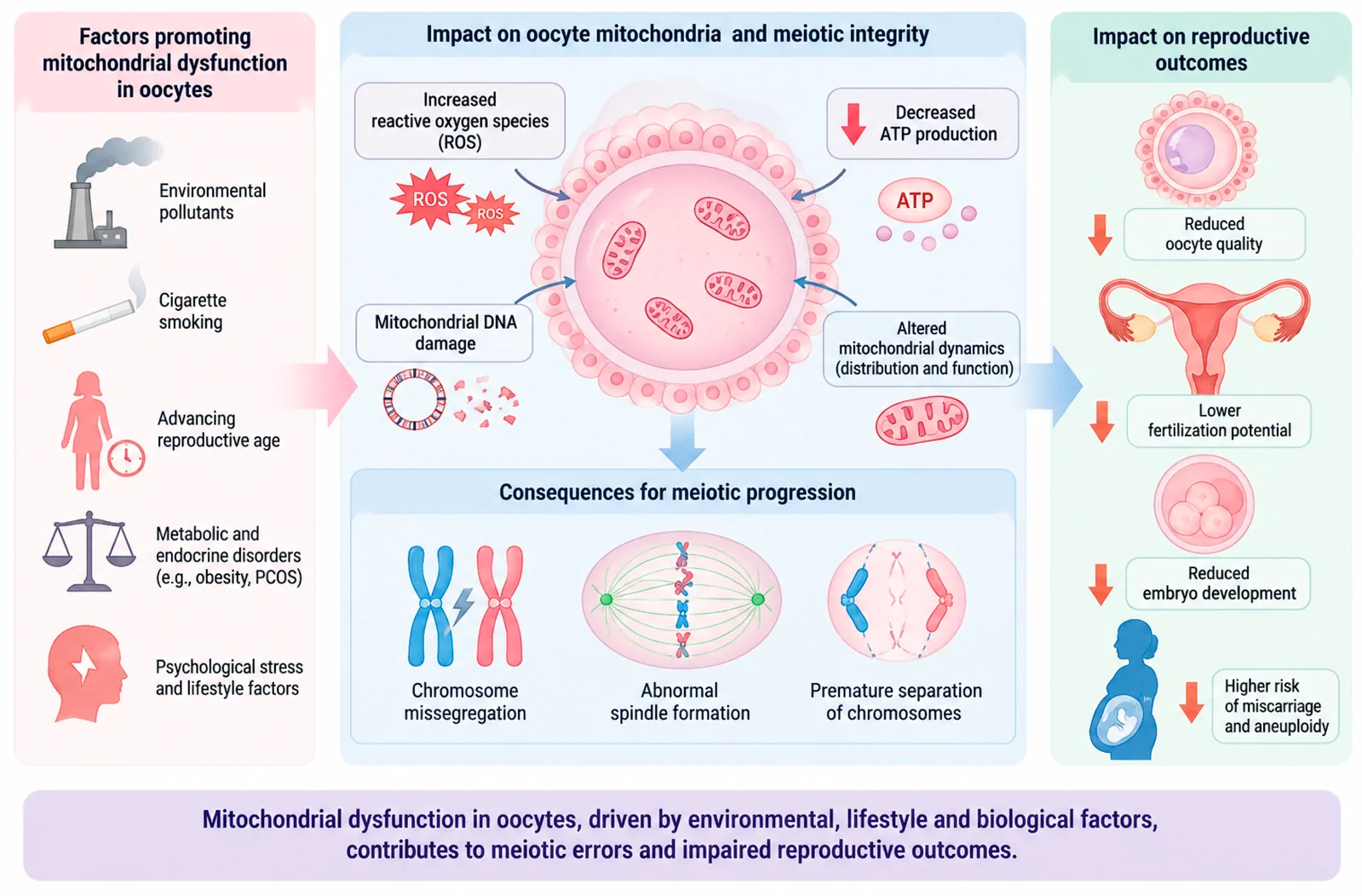
Proposed relationship between mitochondrial dysfunction, meiotic integrity, and reproductive competence during oocyte aging. Advancing reproductive age and environmental and metabolic factors may contribute to mitochondrial dysfunction, characterized by increased reactive oxygen species (ROS), mitochondrial DNA damage, reduced ATP production, and altered mitochondrial dynamics. These alterations may impair spindle organization and chromosome segregation, thereby contributing to reduced oocyte and developmental competence and an increased risk of aneuploidy. Figure created by the authors based on the evidence discussed in Section 3.2.

Experimental studies indicate that severe mitochondrial dysfunction and increased mtDNA mutation burden can impair spindle organization, disrupt chromosome alignment, and increase the risk of meiotic errors (Figure 3) [43,44]. Nevertheless, much of the causal evidence derives from mouse models or experimentally induced mitochondrial dysfunction. Direct evidence demonstrating that naturally occurring mtDNA mutations cause aneuploidy, implantation failure, or miscarriage in humans remains limited.

Collectively, the available evidence most consistently supports impaired bioenergetic function and altered redox homeostasis as age-associated features of declining oocyte competence [45]. In contrast, mtDNA copy number varies considerably across studies and cannot currently be considered a reliable marker of oocyte aging, whereas the extent and functional significance of age-associated mtDNA mutation accumulation in human oocytes remain unresolved, with recent evidence suggesting that purifying selection may limit the persistence of deleterious variants [42,46]. Altered metabolic communication between the oocyte and surrounding follicular cells may further contribute to these changes. The interaction between gonadotropin signaling and mitochondrial metabolism in granulosa and cumulus cells may provide an important mechanistic link between endocrine aging and declining oocyte competence [47].

### 3.3. Telomere Attrition and Telomerase Dysregulation

Telomeres are specialized nucleoprotein structures located at chromosome ends and contain tandem repeats of the DNA sequence (TTAGGG)n. Together with the shelterin protein complex, telomeres protect chromosome ends from degradation, inappropriate DNA-damage responses, and end-to-end fusion. Their length can be maintained in telomerase-expressing cells by telomerase, a ribonucleoprotein enzyme composed of the catalytic subunit telomerase reverse transcriptase (TERT) and the RNA component (TERC), which serves as a template for telomere elongation [48,49,50,51].

Unlike proliferating somatic cells, oocytes remain arrested at prophase I of meiosis for decades without undergoing repeated cell divisions. Therefore, replication-dependent telomere erosion is unlikely to be the principal mechanism of telomere shortening in arrested oocytes [52]. Instead, telomere dysfunction may result from oxidative damage and age-related impairment of telomere-maintenance and protective mechanisms. By contrast, granulosa cells undergo extensive proliferation during follicular development and may experience both replication- and oxidative-stress-associated telomere attrition. Telomeric DNA is particularly susceptible to oxidative damage because of its high guanine content. ROS generated during mitochondrial dysfunction can produce oxidative lesions in telomeric DNA, interfere with telomere replication and repair, and compromise telomere integrity [53]. These processes provide a biologically plausible link between mitochondrial dysfunction, oxidative stress, and altered telomere integrity during ovarian aging.

**Figure 4:**
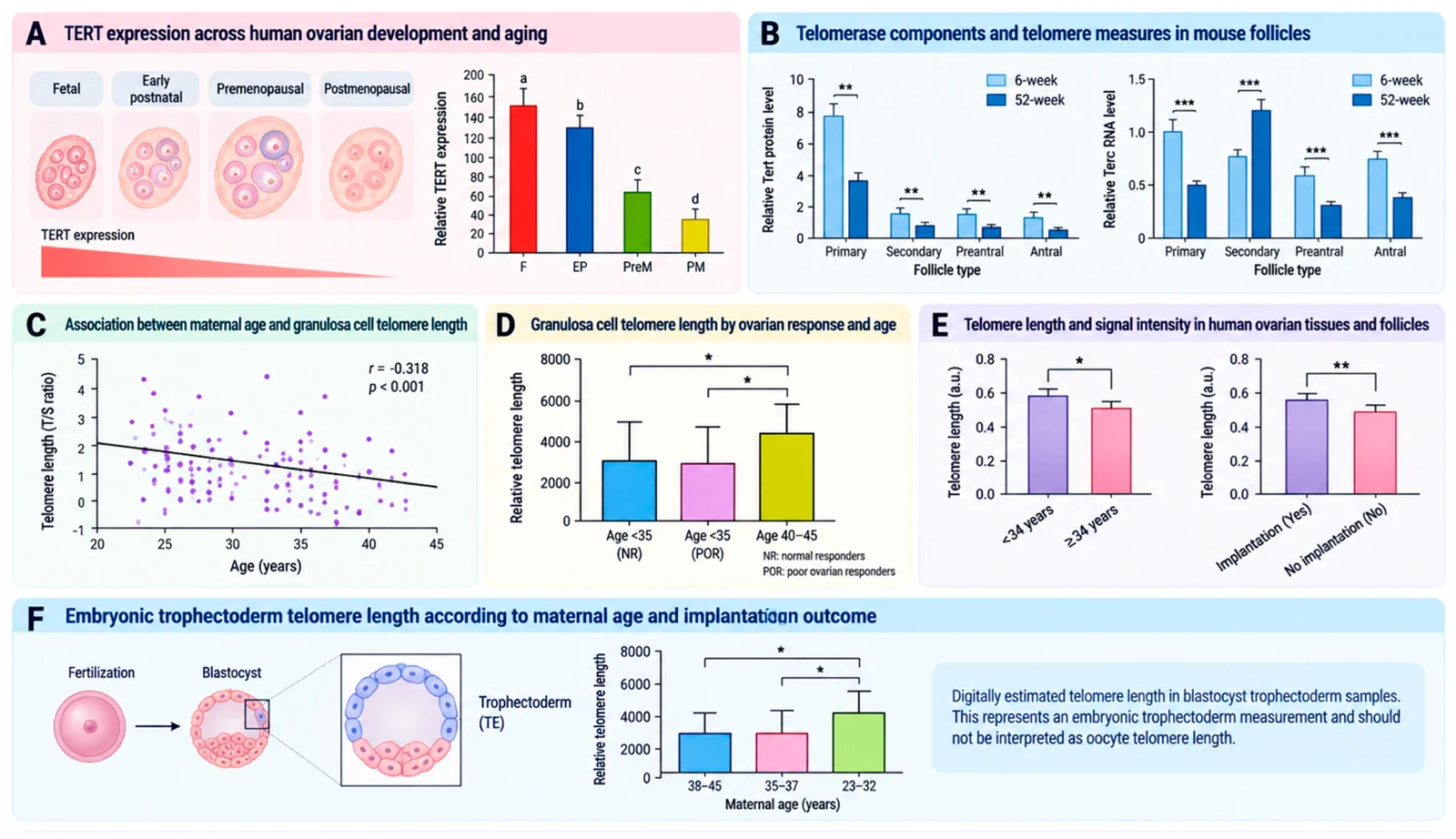
Summary of age- and ovarian-response-associated alterations in telomere biology. (**A**) TERT expression in human ovarian tissues across developmental and reproductive stages, based on Uysal et al. [54]. (**B**) Stage-specific alterations in Tert and Terc expression and telomerase-related measures in follicles from young and aged mice, based on Kosebent and Ozturk [55]. (**C**) Relationship between maternal age and relative telomere length (RTL) in granulosa cells from women undergoing IVF, based on Hao et al. [56]. (**D**) Granulosa-cell telomere length in young normal responders, young poor ovarian responders, and women aged 40–45 years, based on Yung et al. [57]. (**E**) Telomere length and signal intensity in human ovarian tissues and follicles across developmental and reproductive stages, based on Uysal et al. [54]. (**F**) Digitally estimated telomere length in blastocyst trophectoderm samples according to maternal age and implantation outcome, based on Chien et al. [59]. Importantly, panel (**F**) represents an embryonic trophectoderm measurement and should not be interpreted as oocyte telomere length. Figure created by the authors based on the findings of refs. [54,55,56,57,59]. Statistical significance is indicated as follows: *p* < 0.05 (*), *p* < 0.01 (**), and *p* < 0.001 (***). Different lowercase letters indicate significant differences (*p* < 0.05).

Uysal et al. (2021) investigated TERT and telomere-associated proteins in human ovarian tissues, including oocytes and granulosa cells at different developmental stages [54]. TERT expression and telomere signal intensity were lower in premenopausal tissues (32–46 years) and postmenopausal tissues (53–71 years) than in fetal tissues (19–42 gestational weeks) and early postnatal tissues obtained from neonates younger than one month (Figure 4A,E). These findings are consistent with developmental- and age-associated alterations in telomere maintenance. However, TERT expression does not directly demonstrate telomerase enzymatic activity. Furthermore, comparisons among fetal, neonatal, premenopausal, and postmenopausal ovaries may be influenced by marked differences in follicle abundance and tissue composition. The findings should therefore not be interpreted as evidence that telomerase activity declines uniformly within individual oocytes.

Kosebent et al. (2021) examined *Tert* and *Terc* expression, TERT protein abundance, and telomerase activity in follicles isolated from young (6-week-old) and aged (52-week-old) mice (Figure 4B) [55]. The effects of aging varied according to follicular stage rather than showing a uniform decline across all follicle classes. Several telomere-associated genes and TERT protein levels were reduced in follicles from aged mice, whereas secondary follicles exhibited increased *Tert* and *Terc* expression. The authors proposed that this increase might represent a compensatory response associated with the altered hormonal environment of aging, including elevated FSH concentrations. However, the study did not directly test whether FSH caused the observed changes in telomerase-related gene expression. These findings nevertheless raise the possibility of stage-specific interactions between endocrine signaling and telomere maintenance during ovarian aging.

Additionally, Hao et al. (2023) evaluated relative telomere length (RTL) and TERT in granulosa cells from women undergoing IVF [56]. Shorter granulosa-cell RTL was detected in women older than 35 years than in younger participants (Figure 4C). Interestingly, TERT expression was increased rather than reduced in the older group, possibly reflecting a compensatory response to telomere attrition. TERT expression was inversely associated with oocyte yield; however, neither granulosa-cell RTL nor TERT expression was significantly associated with blastocyst formation or clinical pregnancy. The study also identified age-associated alterations in FSH receptor expression, suggesting a potential link between gonadotropin responsiveness and granulosa-cell aging that warrants further investigation. Importantly, these observations reflect telomere biology in granulosa cells and cannot establish corresponding alterations within the oocyte.

Consistent with these observations, Yung et al. (2023) compared telomere length in luteinized granulosa cells from young normal responders, young women with poor ovarian response (POR), and women aged 40–45 years undergoing ovarian stimulation (Figure 4D) [57]. Granulosa-cell telomeres were significantly longer in young normal responders than in young poor responders or older women. No significant difference was detected between the young poor responders and the older participants. This finding suggests that shorter granulosa-cell telomeres may be associated with impaired ovarian response, rather than representing a specific marker of chronological aging. However, the poor-responder group comprised only eight women, and granulosa cells were collected after controlled ovarian stimulation. Therefore, the findings require validation in larger cohorts.

Collectively, these studies suggest an association between reproductive aging, poor ovarian responsiveness, and alterations in telomere-related parameters in ovarian tissue and granulosa cells. However, the available evidence does not demonstrate consistent age-associated telomere shortening across oocytes, granulosa cells, ovarian follicles, and embryos, and telomere alterations in ovarian somatic cells should not be considered direct biomarkers of biological oocyte age.

**Figure 5:**
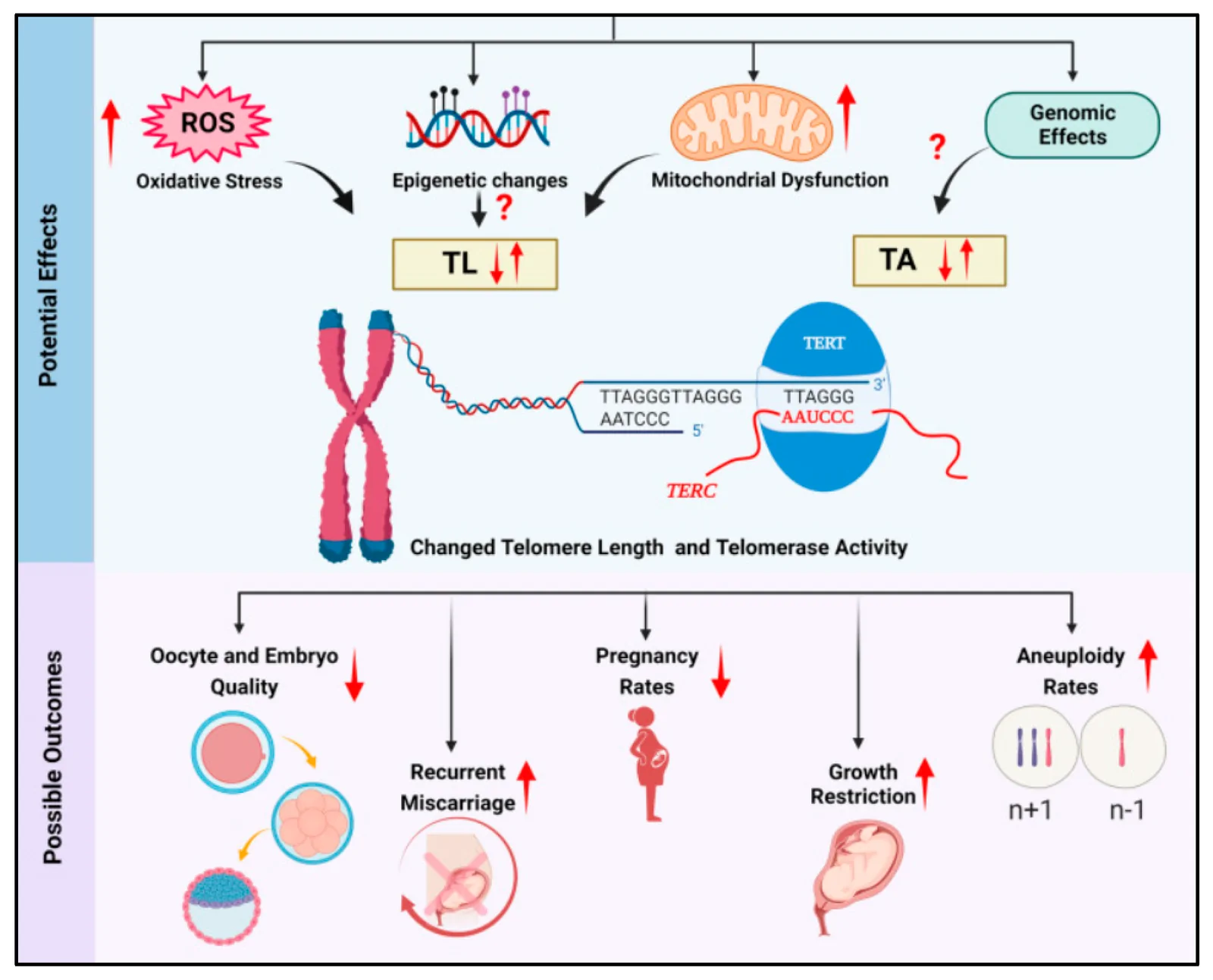
Proposed mechanisms through which telomere dysfunction may affect oocyte quality and reproductive potential. Oxidative damage and impaired telomere maintenance may compromise chromosome-end protection, kinetochore function, spindle organization, chromosome segregation, and subsequent developmental competence. The depicted relationships represent proposed biological associations and mechanisms and should not be interpreted as established causal pathways in humans. Adapted from ref. [50].

Experimental findings suggest a possible mechanistic link between dysfunctional telomeres and meiotic aberrations. Depletion of telomeric repeat-binding factor 1 (TRF1) in mouse oocytes results in impaired kinetochore and centromere organization, defective kinetochore–microtubule attachment and spindle assembly checkpoint function, and premature chromosome separation [58]. These observations provide mechanistic evidence that disruption of telomere-protective machinery can adversely affect meiotic chromosome segregation. However, these findings do not establish natural telomere shortening as a major contributor to age-associated aneuploidy in human oocytes (Figure 5).

Chien et al. (2024) used low-pass whole-genome sequencing data from trophectoderm biopsies to estimate telomere length in human blastocysts [59]. Longer digitally estimated blastocyst telomere length was positively associated with successful implantation, particularly among women aged ≥34 years (Figure 4F). However, telomere length was assessed in blastocyst trophectoderm biopsies rather than in the originating oocytes. Given the extensive remodeling of telomere length during early embryonic development, trophectoderm telomere length and oocyte telomere length represent biologically distinct measurements, and the former should not be interpreted as a surrogate for the latter. These findings therefore do not establish a direct relationship between oocyte telomere length and implantation outcome. In addition, the study was retrospective and conducted at a single IVF center, with a limited number of transferred embryos for which complete outcome data were available. Accordingly, blastocyst telomere length should currently be regarded only as a candidate biomarker of implantation potential rather than as a validated marker of oocyte quality or reproductive aging.

Overall, alterations in telomere biology provide a biologically plausible, but incompletely established, link among oxidative stress, impaired granulosa-cell function, altered chromosome segregation, and reduced developmental competence during reproductive aging. However, most human evidence derives from ovarian somatic cells or other surrogate tissues, and direct evidence of age-associated telomere dysfunction in human oocytes remains limited. Notably, experimental disruption of TRF1 demonstrates that intact telomere-protective machinery is required for normal meiotic chromosome segregation, but it does not establish that physiological telomere shortening during human reproductive aging is a major cause of oocyte aneuploidy or declining developmental competence.

### 3.4. Epigenetic Mechanisms of Oocyte Aging

Epigenetic mechanisms regulate gene activity without altering the underlying DNA sequence and include DNA methylation, histone modifications, and chromatin remodeling. Related epitranscriptomic mechanisms, particularly chemical modifications of RNA, regulate RNA stability, processing, localization, and translation. Together, these regulatory layers are essential for oocyte growth, meiotic competence, fertilization, and early embryonic development [60].

DNA methylation occurs predominantly at CpG dinucleotides and is catalyzed by DNA methyltransferases (DNMTs). DNMT1 primarily maintains existing methylation patterns, whereas DNMT3A, together with its catalytically inactive cofactor DNMT3L, establishes de novo methylation during oocyte growth. DNMT3B also contributes to de novo methylation, although its role in the growing oocyte is less prominent than that of DNMT3A. In oocytes, DNA methylation is enriched across actively transcribed gene bodies, and its functional effects depend on genomic context rather than invariably causing transcriptional repression. Non-CpG methylation is also abundant in oocytes and may contribute to the establishment of the oocyte-specific epigenome. Active DNA demethylation is initiated by ten–eleven translocation (TET) enzymes, which oxidize 5-methylcytosine (5mC) to intermediates that are subsequently converted to unmethylated cytosine [53]. Accumulating evidence indicates that oocyte aging is associated with localized alterations in DNA methylation, reduced epigenomic stability, loss of heterochromatin, and increased transcriptional heterogeneity rather than a uniform change affecting the entire epigenome.

**Figure 6:**
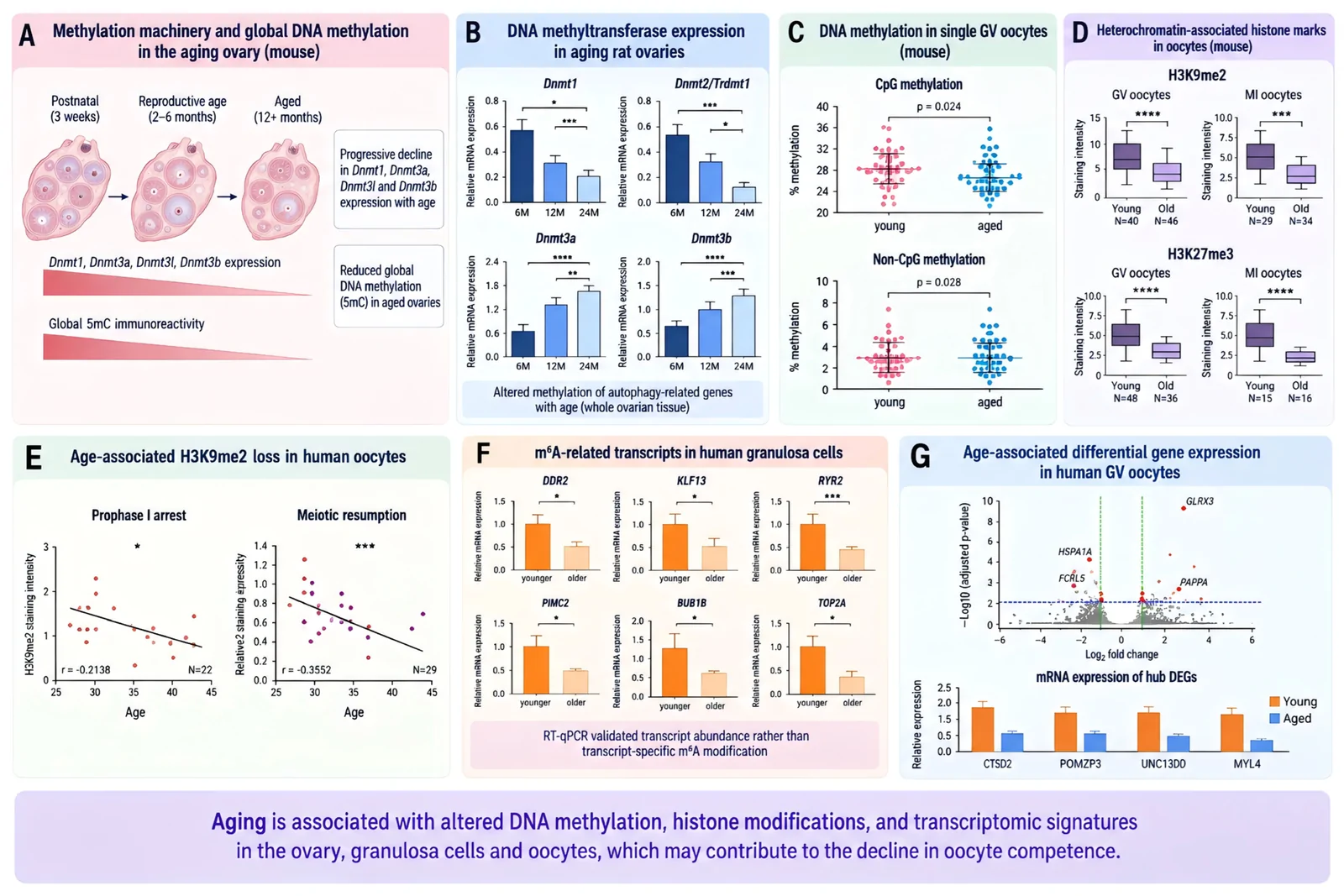
Age-associated epigenetic and epitranscriptomic alterations in ovarian tissue, granulosa cells, and oocytes. (**A**) Schematic summary of age-associated changes in DNA methyltransferase expression and global DNA methylation in whole mouse ovaries, based on Uysal and Ozturk [61]. (**B**) Age-associated alterations in DNA methyltransferase expression and autophagy-gene methylation in whole ovarian tissue from rats, based on Li et al. [62]. (**C**) CpG and non-CpG methylation patterns in single GV oocytes from young and aged mice, based on Castillo-Fernandez et al. [63]. (**D**) Age-associated alterations in the heterochromatin-associated marks H3K9me2 and H3K27me3 in mouse oocytes, based on Wasserzug-Pash et al. [64]. (**E**) Association between reproductive age and H3K9me2 levels in human oocytes, based on Wasserzug-Pash et al. [64]. (**F**) Differential expression of m^6^A-associated transcripts in granulosa cells from women with decreased versus normal ovarian reserve, based on Liu et al. [65]; RT-qPCR validated transcript abundance rather than transcript-specific m^6^A modification. (**G**) Age-associated differential gene expression in human GV oocytes identified by RNA sequencing, based on Orvieto et al. [66]. Figure created by the authors based on the findings of refs. [61,62,63,64,65,66]. Statistical significance is indicated as follows: *p* < 0.05 (*), *p* < 0.01 (**), *p* < 0.001 (***) and *p* < 0.0001 (****).

Uysal and Ozturk examined *Dnmt1*, *Dnmt3a*, *Dnmt3b*, and *Dnmt3l* expression and global 5mC levels in whole postnatal mouse ovaries across young, prepubertal, pubertal, postpubertal, and aged groups [61]. *Dnmt1*, *Dnmt3a*, and *Dnmt3l* expression, together with global 5mC immunoreactivity, declined progressively with age, whereas *Dnmt3b* expression increased in pubertal, postpubertal, and aged ovaries (Figure 6A). The increase in *Dnmt3b* may reflect a compensatory or cell-type-specific response, but this interpretation was not tested directly. Moreover, because the analysis involved intact ovarian tissue, the observed changes may partly reflect the marked age-related alteration in ovarian cellular composition and follicle abundance. The findings therefore cannot be attributed exclusively to oocytes.

Li et al. (2020) analyzed whole ovarian tissues from 6-, 12-, and 24-month-old rats and found that serum estradiol concentrations and the expression of several autophagy-related genes declined with age [62]. *Dnmt1* and *Dnmt2* expression decreased, whereas *Dnmt3a* and *Dnmt3b* expression increased (Figure 6B). The authors also observed increased methylation of the *Atg5* promoter in aged ovarian tissue, accompanied by reduced expression of autophagy-related genes. DNMT2—now commonly designated TRDMT1—is primarily an RNA cytosine methyltransferase rather than a conventional DNA methyltransferase. Consequently, reduced expression should not be interpreted as direct evidence of decreased genomic DNA methylation. The contrasting *Dnmt* expression patterns reported in mouse and rat ovaries emphasize that ovarian aging is not associated with a single, uniform methyltransferase profile. Differences in species, age, endocrine state, tissue composition, and analytical methodology may all contribute to the observed variation.

More direct evidence from isolated oocytes was provided by Castillo-Fernandez et al. (2020) [63]. The authors performed parallel single-cell transcriptomic and methylomic analyses of naturally ovulated GV-stage oocytes from young (12-week-old) and aged (44–54-week-old) mice. Global DNA methylation patterns were broadly preserved, although aged oocytes exhibited a modest reduction in total CpG methylation and an increase in non-CpG methylation (Figure 6C). The characteristic alternating hypermethylated and hypomethylated domains of the oocyte genome remained intact, and germline differentially methylated regions at imprinted genes retained appropriate methylation irrespective of maternal age. The study nevertheless identified localized age-associated differentially methylated regions, most of which showed reduced methylation in aged oocytes.

Aged oocytes also exhibited reduced transcriptomic complexity and greater inter-oocyte variability. However, only a small proportion of age-associated expression differences corresponded to changes in gene-body methylation, suggesting that many transcriptional alterations arose through post-transcriptional or other regulatory mechanisms. These findings support a model of localized and heterogeneous epigenetic remodeling with age rather than global epigenomic failure.

Wasserzug-Pash et al. (2022) investigated heterochromatin integrity in mouse and human oocytes [64]. Aged mouse oocytes (8–10 months) exhibited reduced levels of the constitutive and facultative heterochromatin-associated marks H3K9me2 and H3K27me3 compared with young oocytes (Figure 6D). Heterochromatin loss preceded a marked increase in aneuploidy and was accompanied by increased expression of L1 and intracisternal A-particle retrotransposons, activation of DNA-damage responses, and impaired oocyte maturation. Experimental disruption of heterochromatin in young mouse oocytes reproduced aspects of this phenotype, whereas pharmacological activation or overexpression of heterochromatin-promoting factors partially restored heterochromatin marks and maturation in aged oocytes. Age-associated loss of H3K9me2 was also observed in human prophase-I-arrested oocytes (Figure 6E). These experiments offer more compelling mechanistic evidence than observational methylation studies, supporting a potential causal role for heterochromatin disruption and loss of retrotransposon silencing in reduced oocyte competence. However, whether these mechanisms represent primary drivers of physiological oocyte aging in humans remains to be established.

Beyond DNA and histone modifications, increasing attention has been directed toward N6-methyladenosine (m6A), the most abundant internal modification of eukaryotic mRNA. m6A is deposited principally by a writer complex containing METTL3 and METTL14, removed by demethylases such as FTO and ALKBH5, and interpreted by m6A-binding proteins. By regulating RNA stability, processing, export, and translation, m6A represents an additional epitranscriptomic mechanism with potential relevance to ovarian and oocyte function [60,65].

Liu et al. (2022) performed m6A-enrichment sequencing and RNA sequencing in granulosa cells from younger women aged 25–30 years with normal ovarian reserve and older women aged 40–50 years with decreased ovarian reserve [65]. The analysis involved a small cohort of six women. The older group exhibited a higher number of m6A-modified genes. Among the 435 differentially expressed genes, 58 also showed altered m6A modification. These genes were enriched in pathways including FOXO signaling, adherens junctions, and actin-cytoskeleton regulation. Expression differences involving *BUB1B*, *PHC2*, *TOP2A*, *DDR2*, *KLF13*, and *RYR2* were validated using RT-qPCR (Figure 6F). However, RT-qPCR validated transcript abundance, not the presence or extent of m6A modification on the individual transcripts. Moreover, because the study examined granulosa cells rather than oocytes, these findings reflect age-associated epitranscriptomic remodeling of the follicular somatic compartment and should not be directly extrapolated to oocytes.

Since epigenetic and epitranscriptomic mechanisms regulate gene activity, reproductive aging may also be reflected in altered oocyte transcriptomes. Orvieto et al. (2025) compared RNA-sequencing profiles in GV oocytes from women aged 16–29 years and 38–40 years undergoing planned oocyte cryopreservation [66]. The discovery analysis included only six participants—three in each age group—making the study preliminary. Compared with older oocytes, young oocytes exhibited lower expression of *LINC02087*, *POMZP3*, *LINC02749*, *MYL4*, *AGPAT2*, *GCA*, and *LIMK1*, whereas *CLEC3A*, *ARPP21*, and *CITED2* showed higher expression. Therefore, these expression differences should be reported in the direction observed in the original study. Validation in pooled GV-oocyte samples from additional donors confirmed higher *MYL4*, *POMZP3*, and *LINC02087* expression in older oocytes and higher CITED2 expression in younger oocytes (Figure 6G).

These findings identify candidate age-associated transcripts but do not establish whether the observed differences in expression result from DNA methylation, histone modifications, or other epigenetic mechanisms. In addition, gene-set enrichment analysis did not identify a significant pathway, and the limited sample size restricts generalizability. A larger 2025 single-oocyte study and cross-study analysis found substantial discordance among published human oocyte transcriptomic datasets, with only a relatively small number of genes showing reproducible age-associated changes [67]. Accordingly, no individual transcript identified in the Orvieto et al. study should currently be regarded as a marker of oocyte aging until independently validated in larger single-oocyte cohorts [66,67]. Complementary evidence at the protein level has been provided by Galatidou et al. (2024), who applied single-cell proteomics to human GV and MII oocytes from younger and advanced-maternal-age women [68]. Advanced maternal age was associated with reduced abundance of proteasome and TRiC complex components and other proteins involved in proteostasis and meiosis. These findings extend age-associated molecular alterations beyond the transcriptome and suggest that impaired protein homeostasis may contribute to declining oocyte competence, although the observational design does not establish a causal relationship.

Collectively, current evidence indicates that oocyte aging is associated with localized DNA-methylation changes, heterochromatin loss, retrotransposon derepression, altered RNA modification, and increased transcriptional heterogeneity. The strongest causal evidence currently comes from experimental manipulation of heterochromatin in mouse oocytes, whereas most human evidence is observational and is often derived from granulosa cells or small numbers of surplus IVF oocytes.

### 3.5. The Aging Follicular Microenvironment

The oocyte develops within a specialized follicular microenvironment that critically regulates its growth, maturation, and developmental competence. It includes the surrounding granulosa and cumulus cells, the extracellular matrix (ECM), and follicular fluid, which together provide metabolic substrates, endocrine and paracrine signals, and structural support to the developing oocyte. Granulosa cells extend transzonal projections (TZPs), which are composed primarily of filamentous actin (F-actin), traverse the zona pellucida (ZP), and establish gap-junctional communication with the oocyte. These junctions enable the direct bidirectional transfer of ions, metabolites, and signaling molecules essential for oocyte metabolism, maturation, and developmental competence. Consequently, age-related deterioration of the follicular microenvironment may compromise oocyte competence through alterations in somatic-cell support and intercellular communication, in addition to intrinsic molecular changes within the oocyte [69,70].

**Figure 7:**
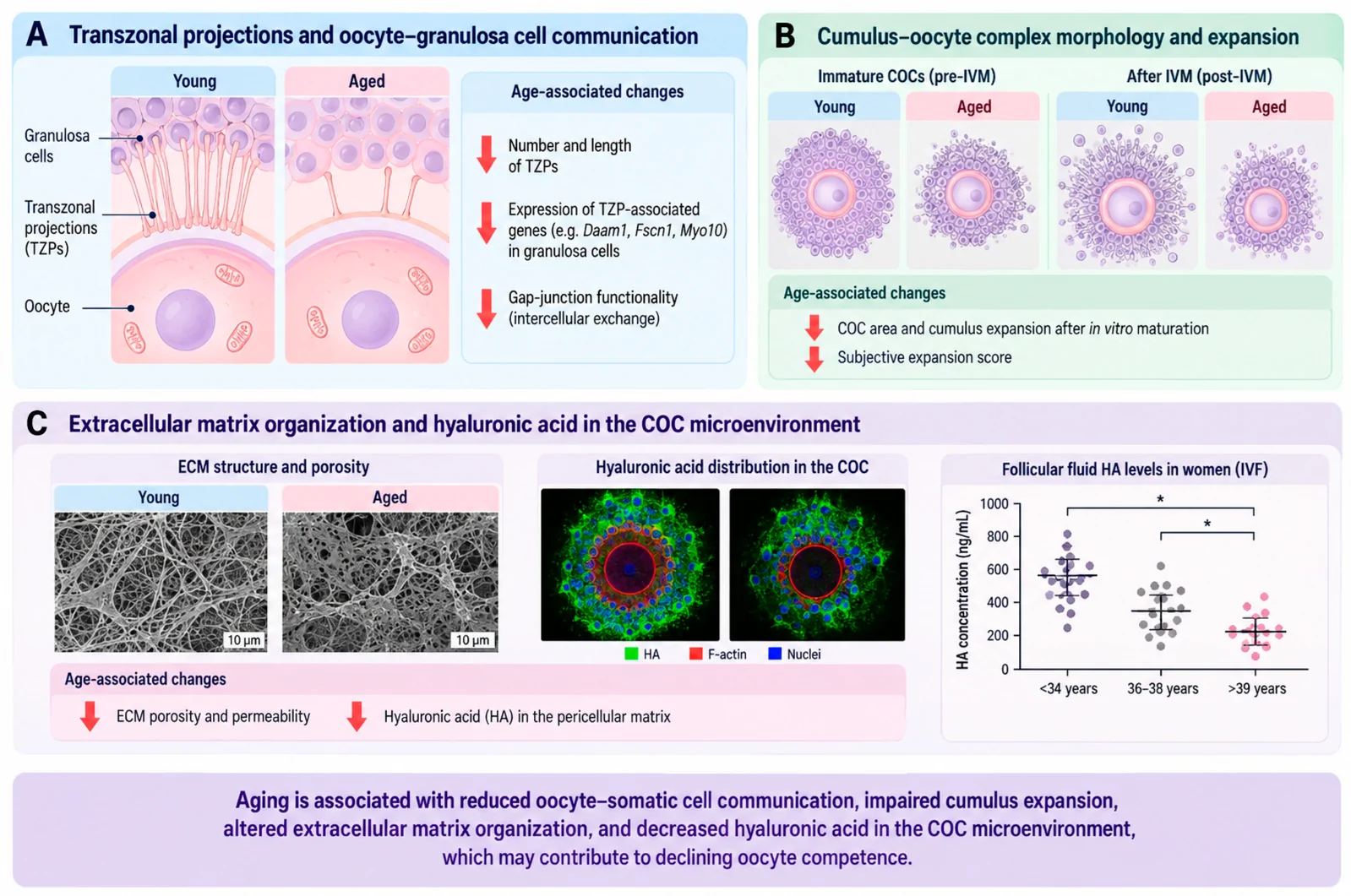
Age-associated alterations in oocyte–somatic cell communication and cumulus–oocyte complex organization. (**A**) Schematic representation of age-associated alterations in transzonal projections (TZPs) and oocyte–granulosa cell communication, including reduced TZP abundance, altered expression of TZP-associated genes (Daam1, Fscn1, and Myo10), and reduced gap-junction functionality. Panel A was created by the authors based on the findings of El-Hayek et al. [71]. (**B**) Cumulus–oocyte complex (COC) morphology, COC area, and cumulus expansion before and after in vitro maturation in young and aged mice. Adapted from ref. [72]. (**C**) Age-associated changes in COC extracellular matrix (ECM) organization and porosity, ECM permeability, and hyaluronic acid (HA) distribution in young and aged mice, together with the association between maternal age and follicular-fluid HA concentration in women undergoing IVF. Adapted from ref. [72]. Statistical significance is indicated as follows: *p* < 0.05 (*).

El-Hayek et al. (2018) investigated age-related changes in TZPs in mouse oocytes. As shown in Figure 7A, aged oocytes (13 months) exhibited significantly fewer TZPs than those from young mice (3 months), indicating impaired structural communication between the oocyte and granulosa cells [71]. Consistent with this finding, RT-qPCR analysis demonstrated a 60–70% reduction in the expression of the TZP-associated genes *Daam1*, *Fscn1*, and *Myo10* in granulosa cells from aged mice (Figure 7A). Furthermore, fluorescence recovery after photobleaching (FRAP) analysis revealed reduced gap-junction functionality in aged mice (Figure 7A). Together, these findings provide experimental evidence that reproductive aging is associated with impaired structural and functional communication between the oocyte and surrounding granulosa cells.

Beyond alterations in intercellular communication pathways, Babayev et al. (2023) investigated age-related changes in the structural organization of the cumulus–oocyte complex (COC) in mice [72]. COCs isolated from young (6–12 weeks) and aged mice (14–17 months) were subjected to in vitro maturation to assess age-associated differences in cumulus expansion during final maturation. Those from young mice exhibited a marked increase in surface area and pronounced cumulus expansion, whereas those from aged mice showed a significantly smaller increase in surface area and remained more compact following maturation (Figure 7B). The authors reported no differences in the number of cumulus cells between age groups, suggesting that the reduced expansion reflected altered cumulus-cell function or extracellular-matrix organization rather than simply a reduction in cell number.

To investigate the mechanisms underlying the age-related decline in cumulus-cell function, Babayev et al. analyzed the morphology of the cumulus extracellular matrix (ECM) and the production of its principal component, hyaluronic acid (HA). Under physiological conditions, the ECM forms a highly organized three-dimensional network that provides structural support for normal cumulus expansion during the final stages of follicular maturation. The ECM of COCs from aged mice exhibited marked structural disruption, characterized by an irregular meshwork, increased porosity and permeability, and reduced hyaluronic acid (HA) production (Figure 7C). These alterations were consistent with the reduced expansion capacity observed in aged COCs. Babayev et al. also reported a decrease in follicular-fluid HA concentration with advancing reproductive age in women undergoing IVF (Figure 7C), providing human evidence that components of the follicular ECM may change with reproductive aging. However, follicular-fluid HA concentration represents an indirect measure of the cumulus ECM and does not establish that the structural abnormalities observed in aged mouse COCs also occur in humans. In addition, the use of samples obtained following controlled ovarian stimulation may influence the follicular microenvironment and should be considered when interpreting these findings.

More direct experimental evidence for the contribution of the follicular microenvironment to age-associated loss of oocyte competence was provided by Wang et al. (2024) [73]. Using a chimeric follicle model, the authors reciprocally transferred young and aged mouse oocytes into follicles from which the native oocyte had been removed. Young oocytes cultured within aged follicles exhibited impaired meiotic maturation and developmental potential, whereas aged oocytes cultured within young follicles showed improved maturation, blastocyst formation, and live-birth rates. These improvements were accompanied by enhanced oocyte–somatic-cell interactions, transcriptomic and metabolomic remodeling, improved mitochondrial function, and greater fidelity of meiotic chromosome segregation. These findings provide experimental evidence that the age of the surrounding follicular environment can directly influence oocyte competence. However, the study was conducted in a mouse model, and whether modification of the human follicular microenvironment can similarly improve the developmental competence of aged oocytes remains unknown.

Beyond the structural and functional changes observed in the follicular microenvironment with advancing age, age-associated metabolic and mitochondrial alterations have also been identified in its somatic-cell compartment. Age-associated mitochondrial dysfunction has been documented in human granulosa cells, while metabolomic analyses have identified alterations in energy and redox metabolism in cumulus cells surrounding oocytes from older women [38,39]. These findings are particularly relevant because cumulus cells provide essential metabolic support to the oocyte. Therefore, age-associated alterations in mitochondrial function, redox homeostasis, and energy metabolism within follicular somatic cells could impair the quality or availability of metabolic support provided to the oocyte. These metabolic alterations may be further compounded by age-associated impairment of TZPs and gap-junctional communication, potentially limiting the exchange of metabolites and regulatory signals between the oocyte and surrounding somatic cells [71].

**Figure 8:**
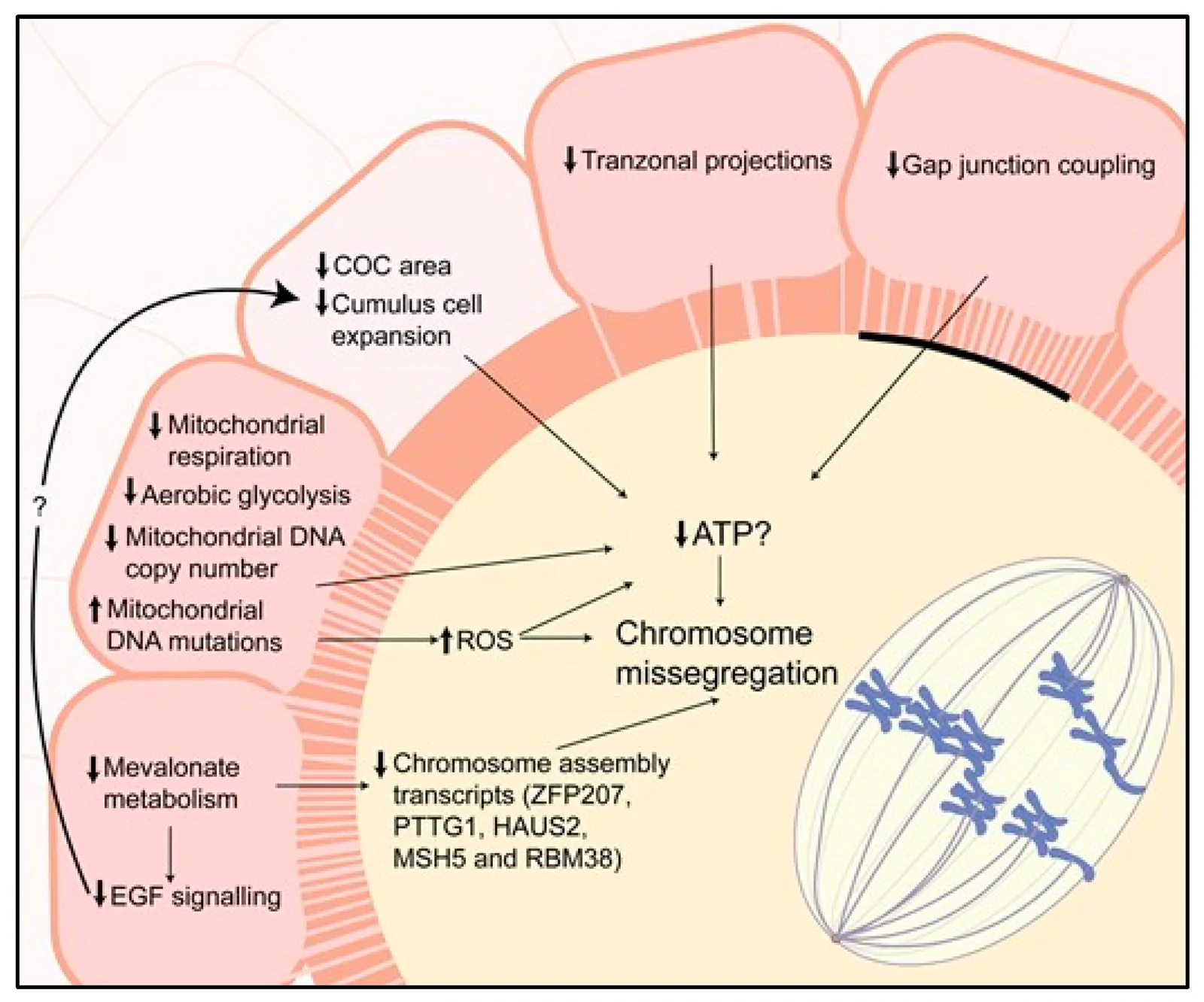
Proposed relationships between age-associated alterations in cumulus-cell function and oocyte competence, including changes in intercellular communication, mitochondrial and metabolic function, and chromosome segregation. The schematic represents a proposed mechanistic framework and should not be interpreted as establishing direct causal relationships in humans. Adapted from ref. [70].

Collectively, existing evidence suggests that reproductive aging alters multiple components of the follicular microenvironment, including oocyte–granulosa cell communication, cumulus expansion, ECM architecture, HA production, and somatic-cell metabolism (Figure 8). Rather than representing isolated abnormalities, these alterations may reflect interconnected mechanisms that collectively impair follicular support of the oocyte. Experimental findings from chimeric mouse follicles further indicate that the age of the surrounding follicular environment can directly influence oocyte developmental competence. Reduced TZP abundance and gap-junction coupling may impair intercellular communication, whereas mitochondrial and metabolic dysfunction in granulosa and cumulus cells may compromise the metabolic support available to the oocyte. Alterations in the cumulus ECM may additionally impair the coordinated expansion and periovulatory remodeling of the COC required for normal ovulation and fertilization. At present, no marker of the age-associated follicular microenvironment has been sufficiently validated for routine use in IVF laboratories. Determining whether these changes represent modifiable contributors to oocyte aging, rather than accompanying features of reproductive aging, will be essential before the follicular microenvironment can be considered a therapeutic target.

The principal studies supporting the molecular and cellular mechanisms of oocyte aging discussed above, together with their major findings and interpretative limitations, are summarized in Table 1.

## 4. Experimental Strategies Targeting Oocyte Competence

The discovery of potentially modifiable pathways involved in oocyte aging has sparked considerable interest in interventions aimed at preserving or improving oocyte competence. Currently, experimental strategies primarily target oxidative stress, age-related metabolic decline, mitochondrial dysfunction, and age-associated alterations in the follicular microenvironment. Nevertheless, the strength of evidence varies substantially among interventions, and most mechanistically promising approaches remain supported primarily by animal or preclinical studies. Importantly, improvements in molecular, metabolic, or cellular markers of oocyte competence should not be interpreted as evidence of delayed oocyte aging or extension of reproductive lifespan.

### 4.1. Antioxidant and Metabolic Approaches

Given the contribution of mitochondrial dysfunction and redox imbalance to reproductive aging, oxidative stress has been extensively investigated as a potentially modifiable pathway. Antioxidants such as coenzyme Q10, vitamins C and E, L-carnitine, melatonin, quercetin, resveratrol, astaxanthin, and ginsenoside Rb1 have been investigated for their potential to reduce ROS production, enhance endogenous antioxidant defenses, and preserve mitochondrial function [74]. Experimental studies have reported beneficial effects of several of these interventions on oxidative-stress markers, mitochondrial function, and oocyte quality. Clinical studies have also evaluated antioxidant supplementation in women undergoing ART. In a randomized trial of young women with decreased ovarian reserve, CoQ10 pretreatment was associated with improved ovarian response and several embryological outcomes, although clinical pregnancy and live-birth rates were not significantly increased [75]. In contrast, a placebo-controlled randomized trial of melatonin supplementation during ovarian stimulation found no significant improvement in oocyte or embryo parameters, clinical pregnancy, or live birth [76]. These findings illustrate the heterogeneous clinical evidence and, importantly, neither study was designed to determine whether antioxidant supplementation delays physiological oocyte aging. Accordingly, current clinical evidence does not establish that antioxidant supplementation preserves reproductive lifespan or delays the age-associated decline in oocyte competence in women.

Targeting age-associated NAD^+^ depletion represents a more specific metabolic strategy. Bertoldo et al. (2020) demonstrated that supplementation with the NAD^+^ precursor nicotinamide mononucleotide (NMN) improved reproductive outcomes in aged mice [77]. NMN treatment restored oocyte NAD^+^ levels and improved several measures of oocyte quality and fertility, with beneficial effects extending to live-birth outcomes. These findings provide preclinical support for a mechanistic link between age-associated NAD^+^ depletion, metabolic dysfunction, and reduced oocyte competence. However, the evidence derives from aged mouse models, and it remains unknown whether comparable NAD^+^ depletion is therapeutically modifiable in human oocytes or whether NMN supplementation can improve clinically meaningful reproductive outcomes in women. Optimal dosing, treatment duration, and long-term reproductive and offspring safety also remain to be established.

### 4.2. Mitochondrial Transfer

**Figure 9:**
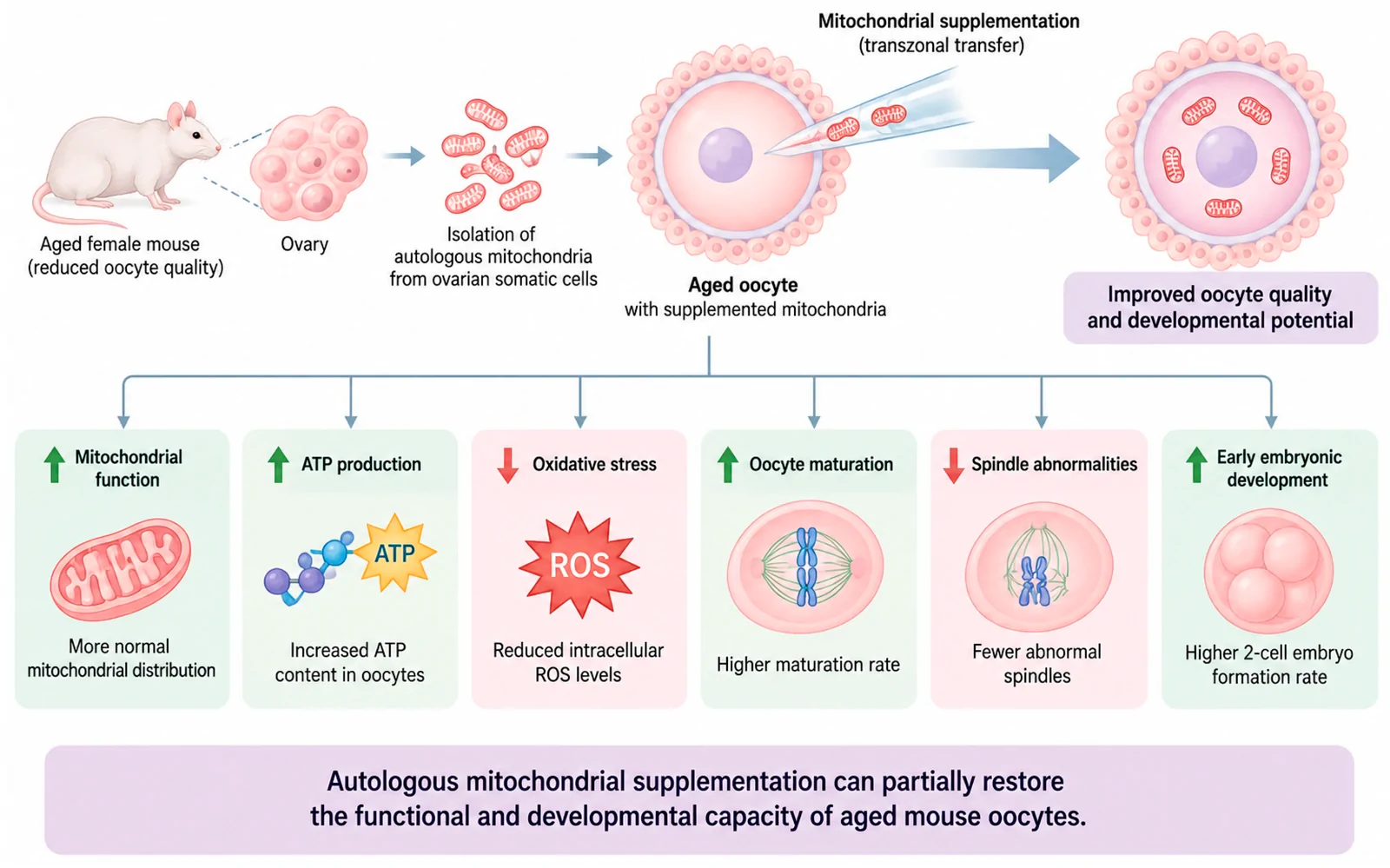
Experimental mitochondrial supplementation as a strategy to improve aged-oocyte function in mice. Autologous mitochondrial supplementation of aged mouse oocytes was associated with improved mitochondrial distribution and function, increased ATP content, reduced intracellular ROS levels, improved oocyte maturation and spindle organization, and enhanced early embryonic development. Figure created by the authors based on the experimental findings reported by Tang et al. [78].

Alternatively, a more direct approach to targeting age-associated mitochondrial dysfunction involves introducing functional mitochondria into aged oocytes with the aim of improving bioenergetic function. Tang et al. (2022) investigated mitochondrial transfer from induced granulosa-like cells (iGCs) to aged mouse oocytes [78]. Co-culture of iGCs with aged oocytes enabled mitochondrial transfer from the iGCs to the oocytes via newly formed transzonal projections (Figure 9).

Following mitochondrial transfer, aged oocytes exhibited improved mitochondrial morphology and function, increased ATP levels, reduced ROS accumulation, and improved spindle organization. These changes were accompanied by improvements in oocyte maturation, fertilization, and early embryonic development (Figure 9) [78]. These findings suggest that supplementation with functional mitochondria can ameliorate specific mitochondrial and developmental abnormalities associated with oocyte aging in this experimental mouse model. Nevertheless, mitochondrial transfer raises several translational concerns, including mitochondrial heteroplasmy, nuclear–mitochondrial compatibility, the persistence and potential transmission of transferred mtDNA, and the long-term reproductive and offspring consequences associated with modification of the germline mitochondrial complement. In addition, the extent to which the TZP-mediated transfer observed in this experimental system can be reproduced or translated to human oocytes remains uncertain. Therefore, these findings should be regarded as preclinical evidence rather than evidence supporting the clinical use of mitochondrial transfer to counteract physiological oocyte aging in women.

### 4.3. Exosome-Based Modulation of the Follicular Microenvironment

An alternative approach involves targeting the follicular microenvironment rather than directly manipulating the oocyte. Extracellular vesicles, including exosomes, mediate intercellular communication by transferring regulatory molecules such as miRNAs, proteins, and other bioactive cargo [79]. In the ovarian follicle, exosomal signaling may therefore represent one mechanism through which surrounding somatic cells influence oocyte development and competence.

Liu et al. (2024) investigated this concept experimentally in aged mice [15]. Exosomes isolated from the follicular fluid of young mice ameliorated several age-associated ovarian alterations, with evidence implicating the miR-320a-3p/FOXQ1 pathway. Exosome treatment in aged mice improved mitochondrial function in granulosa cells, increased ATP production, reduced oxidative stress, and was associated with enhanced follicular development and oocyte quality [15]. These findings provide preclinical evidence that targeting intercellular signaling within the follicular microenvironment can indirectly improve parameters associated with oocyte competence without direct manipulation of the oocyte itself.

Nonetheless, exosome-based interventions remain at an early preclinical stage. Important translational challenges include characterization and standardization of molecular cargo, biodistribution, dose and route of administration, cell-specific targeting, manufacturing reproducibility, and long-term reproductive and offspring safety. In addition, because extracellular vesicles contain heterogeneous molecular cargo that may vary according to their cellular origin, physiological state, and isolation method, the specific components responsible for the observed biological effects remain to be fully defined. Whether comparable effects can be achieved using human follicular-fluid-derived exosomes and whether such interventions can safely improve clinically meaningful reproductive outcomes remain unknown.

Together, these approaches target distinct aspects of the oocyte aging phenotype. Antioxidants target redox imbalance, NAD^+^ replenishment addresses metabolic decline, mitochondrial transfer seeks to restore organelle function, and exosome-based strategies modulate intercellular communication within the follicular microenvironment. Available evidence indicates that several molecular and cellular pathways associated with reproductive aging are experimentally modifiable; however, this does not establish prevention, reversal, or delay of physiological oocyte aging in women.

The principal experimental strategies targeting oocyte aging, their proposed mechanisms, reported effects, and current translational limitations are summarized in Table 2.

## 5. Future Directions and Translational Perspectives

Despite substantial advances in understanding the biology of reproductive aging, several important questions remain unresolved. A major challenge is distinguishing molecular alterations that are causal drivers of declining oocyte competence from those that represent secondary consequences or biomarkers of chronological and ovarian aging [5,80]. Mitochondrial dysfunction, oxidative stress, telomere alterations, epigenetic remodeling, and deterioration of oocyte–somatic cell communication frequently coexist, but their temporal sequence and relative contribution to loss of developmental competence remain poorly defined [81]. Future research should therefore move beyond individual mechanisms of aging and adopt integrated frameworks that examine their interactions during reproductive aging [82,83].

Studies of IVF populations may also be confounded by infertility diagnosis, ovarian reserve, ovarian stimulation, gonadotropin dose, and culture conditions. Future human studies should therefore clearly distinguish chronological age from ovarian reserve and infertility status, while accounting for treatment-related and clinical factors that may influence the molecular phenotype [84,85].

Single-cell and multi-omics techniques offer important opportunities to address some of these limitations. The application of transcriptomics, epigenomics, metabolomics, proteomics, and mitochondrial genomics at the single-oocyte level, together with analysis of matched cumulus or granulosa cells, may help distinguish reproducible molecular signatures of aging from interindividual variability [86]. Moreover, studies integrating oocytes with matched follicular somatic cells and subsequent developmental outcomes may help clarify whether changes in the follicular microenvironment are associated with, or potentially contribute to, intrinsic oocyte defects.

The relationship between endocrine aging and molecular deterioration also warrants further investigation. FSH, AMH, inhibin B, and other endocrine markers are well established for assessing ovarian reserve, but their relationship with the molecular competence of individual oocytes remains less well defined [6]. Future studies integrating gonadotropin-receptor signaling, steroidogenesis, granulosa- and cumulus-cell metabolism, mitochondrial function, and oocyte developmental outcomes may determine whether altered endocrine responsiveness actively contributes to cellular aging or predominantly reflects progressive follicular depletion. Such studies should account for exogenous gonadotropin exposure, particularly when samples are obtained during controlled ovarian stimulation [23,24,25].

From a therapeutic perspective, future research should distinguish between interventions that improve individual markers of oocyte quality and those that produce clinically meaningful reproductive outcomes. Antioxidants, NAD^+^ restoration, mitochondrial interventions, and extracellular vesicle-based approaches have shown promising experimental results. However, improvements in mitochondrial function, reductions in ROS production, or restoration of spindle organization alone are insufficient to establish their reproductive efficacy [87]. Preclinical studies should incorporate fertilization, embryo development, implantation, live birth, and offspring health as relevant outcomes, followed by appropriately designed human studies where scientifically and ethically justified. Long-term follow-up will be particularly important for interventions capable of altering mitochondrial, metabolic, or epigenetic states with potential consequences beyond fertilization and early embryonic development.

The follicular microenvironment may represent a particularly relevant translational target [88,89]. Unlike direct manipulation of the oocyte or its mitochondrial DNA, modulation of metabolism, intercellular communication, extracellular matrix integrity, or paracrine signaling in granulosa and cumulus cells may offer opportunities to improve somatic support of the oocyte without directly modifying the female germline. The safety and durability of such interventions must also be carefully assessed, especially when extracellular vesicles, metabolic modulators, or other biologically active interventions are involved.

The future advancement of precision reproductive medicine will require moving beyond chronological age alone toward multidimensional assessment of oocyte and follicular competence. The identification and validation of biomarkers reflecting mitochondrial function, redox homeostasis, epigenetic state, and follicular-cell communication may eventually support patient stratification and help identify individuals most likely to benefit from specific interventions [90,91,92]. However, no molecular biomarker currently provides a validated measure of biological oocyte age that can replace chronological age or established clinical measures of ovarian reserve. Candidate biomarkers will require validation in independent human cohorts and demonstration of reproducible associations with clinically meaningful outcomes before they can inform therapeutic decision-making [92].

## 6. Conclusions

Oocyte aging is an integrated and multifactorial biological process involving interconnected changes in endocrine regulation, mitochondrial function, redox homeostasis, telomere maintenance, epigenetic regulation, and the follicular microenvironment. Mitochondrial and metabolic alterations are among the most consistently reported features of reproductive aging, whereas the significance of mtDNA copy number, mtDNA mutation burden, and telomere attrition specifically within human oocytes remains uncertain. Emerging evidence also implicates heterochromatin dysfunction, epigenetic and epitranscriptomic alterations, and disrupted granulosa/cumulus–oocyte communication, supporting the concept that oocyte competence reflects both intrinsic cellular integrity and the endocrine, metabolic, and somatic-cell environment in which the oocyte develops.

Experimental interventions targeting oxidative stress, NAD^+^ metabolism, mitochondrial function, and follicular-cell signaling provide preclinical evidence that several pathways associated with reproductive aging are experimentally modifiable. However, current evidence is insufficient to demonstrate that physiological oocyte aging can be prevented, reversed, or delayed in humans. Collectively, the available evidence indicates that oocyte competence in later reproductive life reflects both the intrinsic integrity of the oocyte and the follicular environment that supports its development. At present, neither ovarian reserve markers nor individual molecular or organelle-based measures adequately capture this biological property in women.

## Figures and Tables

**Table 1:** Principal original studies investigating molecular and cellular mechanisms associated with oocyte and follicular aging.

Study	Year	Domain	Model/Material	Study Design	Key Finding	Main Limitation
Smits et al. [38]	2023	Mitochondrial/redox and energy metabolism	Human immature GV/MI oocytes and matched cumulus cells	Age-group comparison using immunofluorescence, targeted metabolomics, and lipidomics	Advanced reproductive age was associated with oxidative damage, altered glutathione balance, reduced NAD^+^ and phospholipid abundance, and broader alterations in oocyte energy metabolism.	Immature oocytes were obtained after high-dose ovarian stimulation, which may have masked age-related differences.
Liu et al. [39]	2017	Mitochondrial function	Human mural granulosa cells	Cross-sectional age-group comparison of 149 women undergoing IVF (<38 vs. ≥38 years)	Advanced maternal age was associated with more abnormal mitochondrial morphology, reduced membrane potential and ATP levels, and decreased ATP5A1 expression, consistent with impaired OXPHOS function.	Limited mGC yield from some participants, particularly older women, prevented all mitochondrial analyses from being performed in every sample.
Yang et al. [40]	2020	mtDNA integrity and redox metabolism	Human oocytes and POLG-mutator mice	Human age-group comparison (≤30 vs. ≥38 years) combined with an experimental POLG-mutator mouse model	Older women had more mtDNA point mutations in oocytes, while experimental mtDNA mutation accumulation in mice impaired female fertility and altered the oocyte NADH/NAD^+^ redox state.	Causal evidence derives from the POLG-mutator mouse model and does not establish that naturally accumulated mtDNA mutations cause age-associated oocyte dysfunction in women.
Boucret et al. [41]	2017	mtDNA integrity	Human immature oocytes and matched cumulus cells	Observational comparison of 53 oocyte–cumulus complexes from 35 women with diminished vs. normal ovarian reserve	No significant difference in mtDNA variant burden was detected between DOR and NOR groups in either oocytes or cumulus cells; de novo variants were more frequent in cumulus cells than in oocytes.	Small sample using immature discarded oocytes, with sequencing unable to detect heteroplasmy below 2%.
Zhang et al. [44]	2023	Mitochondrial function and meiotic integrity	Young and aged mouse MII oocytes	Experimental age-group comparison of young and aged mouse oocytes with assessment of mitochondrial function, spindle assembly, chromosome segregation, and related molecular alterations	Aged oocytes showed reduced maturation, increased aneuploidy and abnormal spindle assembly, together with mitochondrial dysfunction and altered expression of genes related to spindle assembly and mitochondrial function.	Mouse-oocyte evidence; the association between mitochondrial dysfunction and spindle abnormalities does not establish the same mechanism in aging human oocytes.
Uysal et al. [54]	2021	Telomere biology	Human ovarian tissue (fetal, early postnatal, premenopausal, and postmenopausal)	Cross-sectional comparison of ovarian tissue across four age/life-stage groups using Q-FISH and protein expression analysis	Telomere signal intensity and TERT, TRF1, TRF2, and POT1 expression progressively decreased from fetal to postmenopausal ovarian tissue.	Whole ovarian tissue contains multiple cell types and markedly different follicular compositions across life stages, limiting oocyte-specific interpretation.
Kosebent et al. [55]	2021	Telomere biology and telomerase regulation	Isolated ovarian follicles from adult and aged mice	Age-group comparison of primary, secondary, preantral, and antral follicles from 6-week-old (*n* = 19) and 52-week-old (*n* = 12) mice	Aging was associated with follicle-stage-dependent reductions in telomere-associated gene expression and TERT protein, together with alterations in telomerase activity.	Mouse follicle evidence; relevance to telomere regulation in aging human oocytes remains uncertain.
Hao et al. [56]	2023	Telomere biology	Human granulosa cells	Clinical outcome analysis combined with age-group comparison of granulosa-cell RTL and TERT expression in 160 women undergoing IVF/ICSI	Older women had shorter granulosa-cell telomeres and increased TERT expression; TERT expression was inversely associated with oocyte yield, whereas RTL and TERT were not associated with blastocyst formation or clinical pregnancy.	Granulosa-cell RTL and TERT expression are surrogate measures and do not establish corresponding telomere alterations in human oocytes.
Yung et al. [57]	2023	Telomere biology	Human granulosa cells	Three-group comparison of women undergoing IVF: young normal responders (<35 years), young poor responders (<35 years), and older women (40–45 years)	Granulosa-cell telomere length was shorter in young poor responders and older women than in young normal responders, with no significant difference between young poor responders and older women.	Granulosa-cell telomere length is a surrogate measure and does not demonstrate telomere shortening within the oocyte.
Chien et al. [59]	2024	Telomere biology and reproductive outcome	Human blastocyst trophectoderm biopsies	Retrospective single-center analysis of 965 PGT-A blastocysts, including 216 that underwent FET, using WGS-derived telomere-length estimates	Longer digitally estimated blastocyst telomere length was positively associated with pregnancy outcome after FET, whereas mitochondrial copy number was not associated with post-transfer outcome.	Telomere length was estimated in trophectoderm rather than the originating oocyte and therefore cannot be interpreted as a direct measure of oocyte telomere length or aging.
Uysal and Ozturk [61]	2020	DNA methylation	Whole mouse ovaries across postnatal aging	Age-group comparison of mouse ovaries from young to aged stages assessing DNMT expression and global DNA methylation	*Dnmt1*, *Dnmt3a*, and *Dnmt3l* expression and global DNA methylation progressively decreased with age, whereas *Dnmt3b* expression increased.	Whole-ovary analysis cannot distinguish oocyte-specific epigenetic changes from age-related changes in ovarian cellular and follicular composition.
Li et al. [62]	2020	DNA methylation and autophagy	Whole ovaries from aging rats	Age-group comparison of ovaries from 6-, 12-, and 24-month-old rats assessing autophagy-related genes and DNA methylation	Aging was associated with reduced autophagy-related gene expression, increased *Atg5* methylation, decreased *Dnmt1/Dnmt2* expression, and increased *Dnmt3a/Dnmt3b* expression.	Whole-ovary analysis cannot distinguish oocyte-specific changes from age-related alterations in ovarian cellular and follicular composition.
Castillo-Fernandez et al. [63]	2020	DNA methylation and transcriptomics	Individual mouse oocytes from reproductively young and aged females	Single-oocyte joint transcriptomic and DNA-methylome profiling comparing naturally ovulated oocytes from young and aged mice	Aged oocytes showed greater transcriptomic heterogeneity and modestly reduced CpG methylation, while the overall oocyte methylation landscape and imprinting-associated methylation were largely preserved.	Findings derive from mouse oocytes and require validation in human oocytes.
Wasserzug-Pash et al. [64]	2022	Heterochromatin and chromatin integrity	Mouse oocytes and human prophase I-arrested oocytes	Age-group comparison of mouse and human oocytes combined with experimental manipulation of heterochromatin pathways in mouse oocytes	Aging was associated with loss of heterochromatin marks in mouse and human oocytes; in mice, heterochromatin loss was linked to retrotransposon activation, DNA damage, and impaired oocyte maturation.	Causal evidence derives from experimental manipulation in mouse oocytes, whereas the human evidence is observational.
Liu et al. [65]	2022	Epitranscriptomics (m^6^A) and transcriptomics	Human ovarian granulosa cells	Comparative transcriptome-wide m^6^A profiling and gene-expression analysis of granulosa cells from women with decreased versus normal ovarian reserve	Women with decreased ovarian reserve showed altered m^6^A and gene-expression profiles, including enrichment of pathways related to FOXO signaling, adherens junctions, and actin-cytoskeleton regulation.	Granulosa cells rather than oocytes were examined, limiting direct inference about m^6^A alterations in human oocytes.
Orvieto et al. [66]	2025	Oocyte transcriptomics	Human GV oocytes	Exploratory single-oocyte RNA-seq comparison of GV oocytes from 3 younger and 3 older women, with qPCR validation in pooled GV oocytes from additional donors	Age-associated differences in the expression of several transcripts involved in pathways related to chromosomal stability, mitochondrial function, epigenetic regulation, and cellular signaling were identified, with selected differences supported by qPCR.	Very small discovery cohort (n = 6); identified transcripts require independent validation in larger single-oocyte cohorts.
El-Hayek et al. [71]	2018	Oocyte–somatic-cell communication	Mouse oocytes and follicular cells	Experimental mouse study of TZP formation and germline–somatic-cell communication, including comparison of young and reproductively aged females	GDF9 promoted TZP formation, while reproductive aging was associated with reduced TZP number and impaired oocyte–follicular-cell communication.	Evidence derives from mice; equivalent age-associated alterations in TZPs have not been established in human follicles.
Babayev et al. [72]	2023	Cumulus–oocyte complex and extracellular matrix	Mouse cumulus–oocyte complexes and human follicular fluid	Age-group comparison of cumulus expansion and ECM integrity in mice, with assessment of follicular-fluid HA levels in women	Reproductive aging impaired cumulus expansion and ECM integrity in mice, with reduced HA and altered ECM-related gene expression; follicular-fluid HA levels also decreased with age in women.	Mechanistic COC abnormalities were demonstrated in mice, whereas the human evidence was limited to an age-associated difference in follicular-fluid HA.
Galatidou et al. [68]	2024	Proteostasis and meiosis	Human GV and MII oocytes	Single-cell proteomic comparison of oocytes from younger and advanced-maternal-age women using plexDIA mass spectrometry	Advanced maternal age was associated with reduced levels of proteasome and TRiC complex components and other regulators of proteostasis and meiosis in human oocytes.	Observational proteomic differences do not establish that the identified protein alterations causally drive age-associated loss of oocyte competence.
Wang et al. [73]	2024	Follicular microenvironment and oocyte–somatic-cell interactions	Young and aged mouse oocytes in chimeric follicles	Reciprocal oocyte transplantation between young and aged follicles followed by in vitro follicle culture and assessment of developmental competence	Young oocytes cultured in aged follicles showed impaired developmental competence, whereas aged oocytes cultured in young follicles showed improved maturation, blastocyst formation, and live-birth outcomes, accompanied by improved mitochondrial function and chromosome segregation.	Experimental mouse follicle model; whether modifying the human follicular microenvironment can similarly improve aged-oocyte competence remains unknown.

**Table 2:** Experimental strategies targeting molecular and cellular mechanisms associated with oocyte aging. Abbreviations: ART, assisted reproductive technology; ATP, adenosine triphosphate; CoQ10, coenzyme Q10; NAD^+^, nicotinamide adenine dinucleotide; NMN, nicotinamide mononucleotide; ROS, reactive oxygen species.

Strategy	Target/Mechanism	Model/Material	Main Effect	Main Limitation	References
Antioxidant supplementation	Oxidative stress, ROS accumulation, and mitochondrial dysfunction	Animal models and women undergoing ART; CoQ10, melatonin, and other antioxidants	Preclinical studies report improved redox and mitochondrial parameters; human trials of CoQ10 and melatonin have shown mixed effects on ovarian response and oocyte, embryo, and reproductive outcomes	Heterogeneous interventions, populations, doses, and outcomes; clinical studies do not establish that antioxidant supplementation delays physiological oocyte aging in women	[74,75,76]
NAD^+^ repletion (NMN)	Age-associated NAD^+^ depletion and metabolic dysfunction	Aged mice	Restored NAD^+^ levels and improved oocyte quality, fertility, and live-birth outcomes	Evidence derives from an aged-mouse model; human efficacy, optimal dosing, and long-term reproductive and offspring safety remain undetermined	[77]
Mitochondrial transfer	Mitochondrial dysfunction and impaired bioenergetics	Aged mouse oocytes	Increased ATP, reduced ROS, and improved mitochondrial function, spindle organization, maturation, fertilization, and early embryonic development	Preclinical approach; heteroplasmy, mitochondrial–nuclear compatibility, germline transmission, procedural standardization, and long-term offspring safety remain unresolved	[78]
Follicular-fluid-derived exosomes	Granulosa-cell mitochondrial function and follicular intercellular signaling	Aged mice	Improved granulosa-cell mitochondrial function, increased ATP, reduced ROS, and improved follicular development and oocyte quality through miR-320a-3p/FOXQ1 signaling	Early preclinical evidence; cargo heterogeneity, biodistribution, dosing, manufacturing standardization, target specificity, and long-term reproductive safety remain unresolved	[15]

## Data Availability

No new data were created or analyzed in this study. Data sharing is not applicable to this article.

## Appendix A

**Table A1:** Representative PubMed search strategies used for the principal thematic domains of the narrative review. Search terms were combined according to the thematic domain under investigation; the strategies shown reflect representative domain-specific combinations rather than a single standardized search string. Abbreviations: AMH, anti-Müllerian hormone; FSH, follicle-stimulating hormone; LH, luteinizing hormone; m6A, N6-methyladenosine; mtDNA, mitochondrial DNA; NAD^+^, nicotinamide adenine dinucleotide; NMN, nicotinamide mononucleotide; ROS, reactive oxygen species; TERT, telomerase reverse transcriptase.

Thematic Domain	Representative PubMed Search Strategy	Search Period/Last Update
Gonadotropin and intraovarian signaling	(“oocyte aging” OR “ovarian aging”) AND (“gonadotropin signaling” OR “follicle-stimulating hormone” OR FSH OR “luteinizing hormone” OR LH OR “anti-Müllerian hormone” OR AMH OR steroidogenesis)	1 January 2017–31 December 2025; targeted update through 17 August 2026
Mitochondrial function, energy metabolism, and oxidative stress	(“oocyte aging” OR “ovarian aging”) AND (mitochondria OR “mitochondrial dysfunction” OR “mitochondrial DNA” OR mtDNA OR “oxidative stress” OR “reactive oxygen species” OR ROS OR “energy metabolism”)	1 January 2017–31 December 2025; targeted update through 17 August 2026
Telomere biology	(“oocyte aging” OR “ovarian aging”) AND (telomere OR telomeres OR telomerase OR TERT OR “telomere attrition”)	1 January 2017–31 December 2025; targeted update through 17 August 2026
Epigenetic mechanisms	(“oocyte aging” OR “ovarian aging”) AND (epigenetic OR “DNA methylation” OR heterochromatin OR transcriptome OR transcriptomics OR “m6A” OR “N6-methyladenosine”)	1 January 2017–31 December 2025; targeted update through 17 August 2026
Follicular microenvironment	(“oocyte aging” OR “ovarian aging”) AND (“follicular microenvironment” OR “granulosa cells” OR “cumulus cells” OR “oocyte-somatic cell communication” OR “follicular fluid”)	1 January 2017–31 December 2025; targeted update through 17 August 2026
Experimental strategies	(“oocyte aging” OR “ovarian aging”) AND (antioxidants OR “coenzyme Q10” OR melatonin OR NAD^+^ OR NMN OR “mitochondrial transfer” OR exosomes)	1 January 2017–31 December 2025; targeted update through 17 August 2026
